# Non-prescribed antibiotic use for children at community levels in low- and middle-income countries: a systematic review and meta-analysis

**DOI:** 10.1186/s40545-022-00454-8

**Published:** 2022-09-30

**Authors:** Dumessa Edessa, Nega Assefa, Yadeta Dessie, Fekede Asefa, Girmaye Dinsa, Lemessa Oljira

**Affiliations:** 1 School of Pharmacy, College of Health and Medical Sciences, Haramaya University, Harar, Ethiopia; 2 School of Nursing and Midwifery, College of Health and Medical Sciences, Haramaya University, Harar, Ethiopia; 3 School of Public Health, College of Health and Medical Sciences, Haramaya University, Harar, Ethiopia; 4 Center for Biomedical Informatics, Department of Pediatrics, College of Medicine, University of Tennessee Health Science Center-Oak Ridge National Laboratory (UTHSC-ORNL, Memphis, TN USA; 5 School of Population and Public Health, University of British Columbia, Vancouver, Canada

**Keywords:** Community-level, Non-prescribed, Antibiotic use, Children, Low- and middle-income countries

## Abstract

**Background:**

Non-prescribed antibiotic use is an emerging risky practice around the globe. An inappropriate use involving nonprescription access is one cause of the rapid increase in antibiotic resistance. Children commonly encounter many self-limiting illnesses for which they frequently use antibiotics without prescription. However, no specific and conclusive evidence exists to inform actions against this unsafe practice. We thus aimed to estimate the pooled proportion of non-prescribed antibiotic use for children at community levels in low- and middle-income countries.

**Methods:**

A systematic search of records was conducted from PubMed/Medline, Embase, Scopus, CINAHL, and Google scholar. Eligible English-language publications were original articles which reported on community-based non-prescribed antibiotic use for children and conducted in low- and middle-income countries. Study features and the number of antibiotics used without prescriptions were extracted and pooled for effect sizes employing a random-effects model. The pooled proportion of non-prescribed antibiotic use was estimated as a percentage.

**Results:**

In this analysis, we included a total of 39 articles consisting of 40,450 participants. Of these, 16,315 participants used non-prescribed antibiotics. The pooled percentage for this use of non-prescribed antibiotics was 45% (95% CI: 40–50%). The estimate was considerably higher in studies involving simulated patient methods (56%; 95% CI: 49–62%) than those studies with community surveys (40%; 95% CI: 34–46%) (P = 0.001). It was also varied by the recall period of antibiotics use—56% (95% CI: 50–62%) for instantly observed practice, 36% (95% CI: 22–50%) for within two week recall, 35% (95% CI: 26–45%) for 1–6 months recall, and 46% (95% CI: 37–54%) for more than six months recall (*P* = 0.001). Primary access points for the non-prescribed antibiotic uses were retail drug outlets.

**Conclusions:**

We found that nearly half of the antibiotics used for children in community settings were without prescriptions. For these unsafe practices, caregivers accessed antibiotics mainly from drug outlets. Hence, context-specific educational and regulatory interventions at these outlets and the community levels are the first steps to improving antibiotic usage for children in low- and middle-income countries.

*Trial registration number:* CRD42021288971 (PROSPERO). https://www.crd.york.ac.uk/prospero/display_record.php?ID=CRD42021288971.

**Supplementary Information:**

The online version contains supplementary material available at 10.1186/s40545-022-00454-8.

## Background

Antimicrobial resistance (AMR) has become an emerging threat to the contemporary world, with an estimated 10 million deaths annually by 2050 [1]. The widespread and inappropriate use of antibiotics in the forms of nonprescription and leftover accesses are common reasons for the rapid increase in resistance to these drugs around the globe. A recent scoping review showed that 62% of the global communities’ antibiotic use was without prescriptions (i.e., nonprescription use) [2]. Other similar studies also revealed the pooled non-prescribed antibiotics use was 66% in high-income countries [3] and 69% in low-income countries [4]. Besides, a systematic review and meta-analysis from low- and middle-income countries (LMICs) showed that non-prescribed antibiotic use ranges from 50% to 93.8%, with a pooled estimate of 78% [5]. This widespread non-prescribed use of antibiotics, because of the high prevalence of infections in the LMICs, puts the setting at a higher risk of developing antibiotic resistance (ABR) than the other settings [6]. Scholars predicted this risk of ABR to be the worst in poorer countries alongside the widespread use of antibiotics for the higher prevalence and emerging infections they usually encounter [7], signaling an urge to the global community towards appropriate antibiotic use.

Antibiotics are prescription-only medicines. However, the public might use them without prescriptions [8]. Several factors that drive the habits of using antibiotics without prescription might include, but are not limited to, the low severity of the illness, accessibility, affordability, and healthcare-seeking behaviors [9]. The usual access points for antibiotic use without prescriptions at the community levels are retail drug outlets and home-stored leftovers [8, 10, 11]. The most common illnesses for which consumers frequently self-prescribe antibiotics include fever, cough, acute upper respiratory tract infections, and diarrhea [4]. Other self-limiting diseases are also common symptoms that may lead to the use of antibiotics without prescriptions [9]. Children are most commonly affected by these self-limiting illnesses for which non-prescribed antibiotics, including watch group ones, are usually sought from retail drug outlets [12]. Users conveniently access the retail drug outlets for timely treatment of some less severe illnesses in resource-limited settings, where basic primary healthcare accesses are inadequate [13]. The retail drug outlets might consider this supply of antibiotics without prescriptions as their public health role. However, the practice is illegitimate, inappropriate, and untargeted because the illness diagnosis is not yet objectively confirmed. It also increases the chance of ABR development [14]. Some of these antibiotics used in this manner are for indications that, in principle, do not require antibiotics [15]. Accordingly, non-prescribed antibiotic use must become the usual choice of providers and consumers for most self-limiting illnesses [16]. As a result, retail drug outlets dispense above two-thirds of the antibiotics that consumers request without prescriptions [17]. Since children have more frequent healthcare visits for treatments of their common illnesses [18], illegitimate antibiotic access on the grounds of these diseases can lead to inappropriate uses and resistance development, which is an emerging threat to public health. This practice of antibiotic use can also involve watch group antibiotics disregarding the World Health Organization’s (WHO) restriction on free access to these drugs at the community levels [12]. According to the WHO, watch group antibiotics are the antibiotic classes at relatively high risk of bacterial resistance selection. They should get the priority of stewardship programs and monitoring [19].

Despite the public and ethical concerns about non-prescribed antibiotic use for children, there is no systematic and comprehensive estimate of this unsafe practice at the global and regional levels. Besides, majorities of the available individual studies are less powered and non-conclusive [20–25]. Most of these individual studies are not sufficiently rigorous to advise and convince program and policy decisions. The available systematic reviews and meta-analyses at global and regional levels regarding non-prescribed antibiotic uses are not specific to children [4, 5, 8]. Accordingly, there is a need for reliable and comprehensive evidence on the appropriateness of antibiotic exposure and use for children that informs policy decisions and context-oriented interventions. We thus aimed to estimate the pooled proportion of community-based non-prescribed antibiotic use for children by caregivers in low- and middle-income countries.

## Methods

The execution of this study followed the statement guidance on the Preferred Reporting Items for Systematic Reviews and Meta-Analysis (PRISMA) [26]. An additional file shows a completed PRISMA checklist in more detail (see Additional file 1). The methodology for this study was pre-specified in a protocol registered in the International Prospective Register of Systematic Reviews (Registration Number: CRD42021288971) [27].

### Data search strategy

We undertook systematic searches of electronic registers and databases on PubMed, Medline, Embase, CINAHL, Scopus, and Google Scholar to identify and include potential literature. Initially, we performed these literature searches from October 21–30, 2021. We also conducted a final update on the literature search in July 2022. Our search strategy involved a combination of one or more of the following terms: “anti-infective” (MeSH), “antibiotic”, “nonprescription” (MeSH), “inappropriate”, “leftover”, “pharmacies” (MeSH), “drug outlet” and “child” (MeSH). We employed Boolean operators (AND, OR) as appropriate alongside these search terms to identify and include more records for the search in question.

### Eligibility criteria

We applied several inclusion and exclusion criteria that the investigating team defined a priori to the records identified. We included original studies conducted anywhere around the globe that addressed non-prescribed antibiotic use for children aged 0–13 years at community settings. It included self-medication with antibiotics from retail drug outlets or pharmacies, private clinics, and leftover uses from home-stored and previously prescribed antibiotics. The studies excluded were abstracts with unrelated data, papers published in languages other than English, and publications without original data (i.e., reviews, correspondence, guidelines, letters, and editorials). Besides, we excluded original articles with reports of insufficient or irrelevant information, case reports, case series, and qualitative studies.

### Study selection

First, the initial data retrieved through a systematic search of electronic databases and registers were identified, downloaded, and linked to the Endnote reference software version 8.2 (Thomson Reuters, Stamford, CT, USA) with the appropriate or compatible formats. Next, we imported the retrieved records from Endnote to the Covidence systematic review software (Veritas Health Innovation, Melbourne, Australia. Available at www.covidence.org). Using the Covidence platform, we detected and marked duplicate records. Due to variation in citation styles of some databases and indexing interfaces, we manually identified and addressed the remaining duplicates of such incompatibilities. In the subsequent steps, two reviewers independently screened the potential articles by titles and abstracts based on the predefined inclusion criteria. Finally, the two reviewers collected and evaluated full texts of the retained articles for eligibility and quality assessments. The reviewers discussed and solved their voting conflicts regarding the screening and eligibility assessments before a final decision. For the final inclusion in this study, we considered all articles that met the eligibility and quality assessments.

### Data extraction

We employed a data collection format prepared in a Microsoft excel sheet to extract all relevant data for the study. Parameters for the data extraction included the name of the first author, the year of the publication, the study setting/country (along with WHO region and World Bank’s income category), the study design, the children’s age, and the childhood condition for which the antibiotics sought. We also considered the primary source of antibiotic access (i.e., drug outlets, clinics, leftovers stored at home), the time duration of antibiotic use recall, the sample size, and the outcome of interest (i.e., the magnitude of non-prescribed antibiotic use for children) as additional parameters for the data extraction.

### Quality and risk of bias assessments

We employed the Joanna Briggs Institute’s (JBI’s) critical appraisal checklist for studies reporting prevalence data to appraise the methodological quality of the retained studies by two independent researchers. For ease of evaluation, we ranked the methodological quality of the studies based on the total number of appraisers’ positive scores marked as ‘yes’ to the appraisal questions. Accordingly, all studies with average positive scores of appraisers added to 60% or above (as moderate or high-quality articles) were considered for the systematic review and meta-analysis.

The risk of bias assessment was also conducted by two reviewers using Hoy et al. appraisal tool for prevalence studies [28]. The appraisal tool contains ten items that assess the risk of bias. The two appraisers solved any point of disagreement between them through discussion. They rated their response to each item with two standard answer options (i.e., the high risk of bias scored as ‘1’ and the low risk of bias marked as ‘0’). To summarize the risk of bias in the studies, we considered participant selection (selection bias), data collection (information bias), outcome measurement (measurement bias), statistics parameters, and other sources of bias. Accordingly, by the appraisal process we classified the potential biases in different sections of the studies into four domains—D1: biases arising from the study participant selection process; D2: the bias linked to the data collection process; D3: the bias in the measurement of the outcome; and D4: biases due to statistics parameter [29]. We rated the risk of bias judgment for the studies with three options based on the summary of answers for all items. Finally, we rated the risk of bias for each study as low for 0–3 scores, with some concerns for 4–6 scores, and high for 7–10 scores [28–30].

### Data analysis and synthesis

We estimated the percentage rate of non-prescribed antibiotic use for children accessed by modes of over-the-counter purchase and sharing of home-stored leftover antibiotics as the primary outcome variable. With the analysis, we sub-grouped the estimate by the study methods (i.e., simulated patient method versus cross-sectional community survey); the recall period of uses requested at the time of data collection (i.e., instantly observed during the data collection versus the use history); the primary sources of antibiotics access; and the type of common childhood illness for the antibiotic use. We also performed subgroup analysis by children’s age and region (continent) of the country where the non-prescribed antibiotic use for children was assessed and reported.

We performed statistical pooling of the estimate for the proportion of non-prescribed antibiotic use according to the random-effects model with generic inverse-variance methods using Stata 14.0. The random-effects model was assumed since the studies identified were observational and had clinical and methodological variabilities across the different sampling frames [31, 32]. We employed a forest plot to present the pooled percentage rate for non-prescribed antibiotic use for children. Herewith, we also performed the heterogeneity assessment, subgroup analyses, and tests for publication bias. In line with this, the essence of subgroup analyses was to explain some features of users which might account for the differences in the effect sizes of non-prescribed antibiotic use [33, 34].

We assessed the publication bias (or small-study effects) using asymmetry inspection with a graph. In this regard, the combined studies met two of the three criteria requirements for employing a funnel plot (i.e., the number of included studies was above ten, and the ratio of maximal and minimal variance across the studies was above four). However, the third criterion of heterogeneity of less than 50% was not fulfilled [35]. As a result, the Doi plot was employed for visual inspection of asymmetry alongside Egger’s regression *p*-value to assess the presence of publication bias [36, 37]. Besides, we created a standard risk of bias graph with a summary plot using the risk of bias visualization (robvis) tool. The robvis tool is a web platform designed for visualizing the risk of bias assessments performed [30]. With the robvis platform, we employed a dataset template to assess the quality of diagnostic accuracy studies (QUADAS) to accommodate the four bias domains considered for the risk of bias appraisal [29]. Lastly, all statistical tests were considered significant for P-values less than 0.05.

## Results

### Study selection

**Fig. 1:**
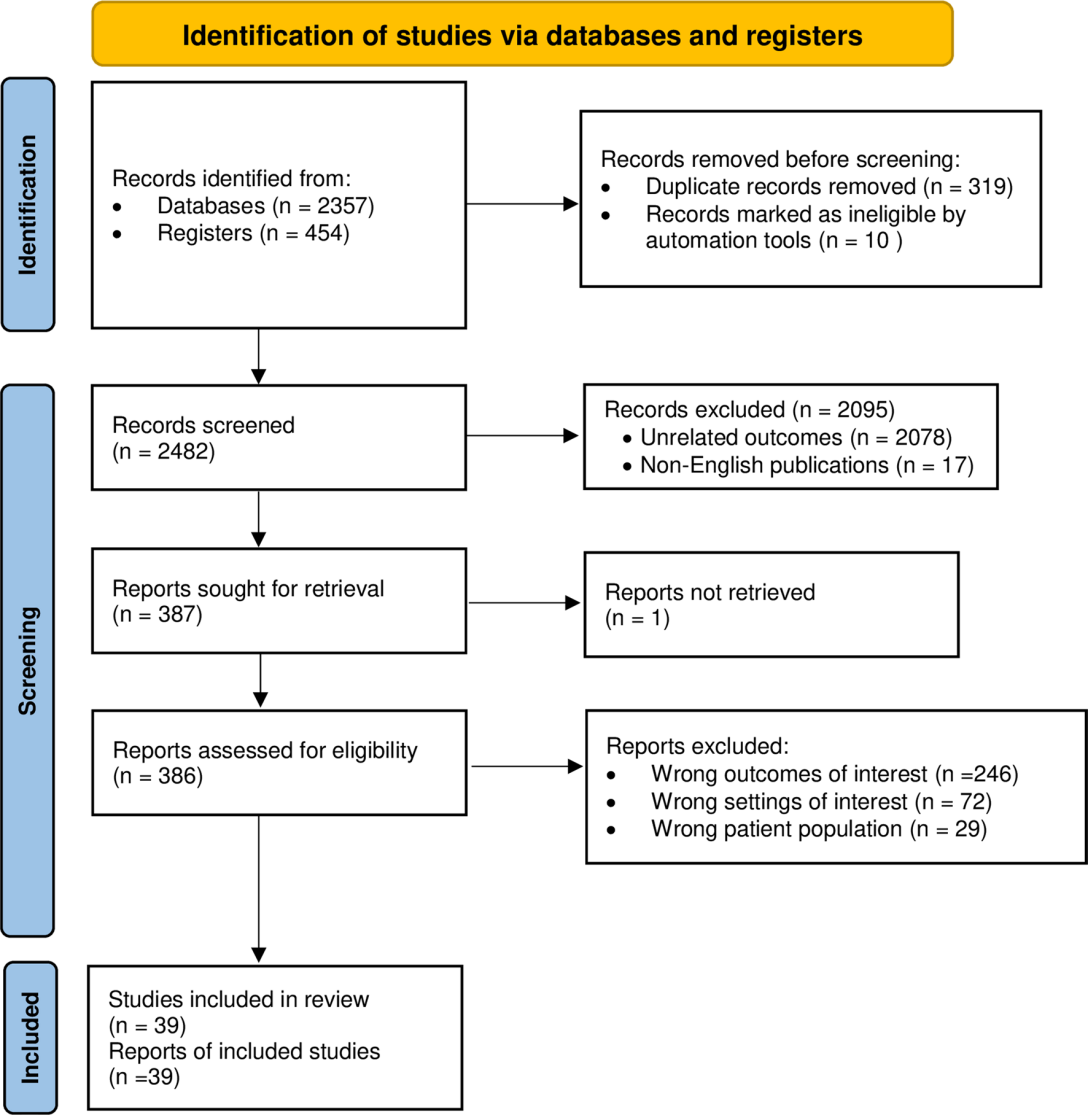
PRISMA 2020 flow diagram depicting the selection process

Through the searches of electronic databases and registers, we identified 2811 records. We retrieved 2801 literature because the electronic register (i.e., Google scholar) marked ten records as ineligible. This means that, ten of the initially identified studies in the Google scholar search were omitted while downloading and linking to the Endnote. After removing 319 duplicates, 2482 records became eligible for screening the titles and abstracts. A total of 2095 records were excluded by screening for titles and abstracts. Of these, 2078 studies had unrelated outcomes of interest, and 17 studies were published in non-English languages. We sought full texts of the remaining 387 records and retrieved 386 papers, but one of the records was a citation link with incomplete information that is not retrievable. We conducted an eligibility assessment for 386 full texts. We excluded 347 of the studies. Of these, 246 studies had wrong outcomes of interest, with wrong setting (i.e., facility-based prescription antibiotic uses) and patient population (i.e., non-prescribed drug uses not specific to children) in 72 and 29 of the remaining studies, respectively. We also evaluated the methodological quality of the retained studies using the critical appraisal checklist of JBI for studies reporting prevalence data. Figure 1 presents the PRISMA flow diagram depicting details of the study selection. Finally, we included 39 studies with methodological quality of moderate to a high percentage as per the average positive responses of the reviewers. An additional file shows the methodological quality status for 39 of the included studies in more detail (see Additional file 2).

### Study characteristics

The 39 studies we included assessed the community-level non-prescribed antibiotic use for children by participating in a total of 40,450 children/caregivers. Of these, 16,315 caregivers used antibiotics for children without prescription, which included leftovers. Publication dates for the studies included in this review were between 2010 and 2022. Sample sizes for the studies range from 73 to 9838, where these two studies with the lowest and the largest sample sizes were conducted in China [25, 38]. The parents or caregivers in the 26 studies used non-prescribed or leftover antibiotics for under-five children [20–25, 39–59], while the parents or caregivers in the remaining 13 studies used antibiotics for children under 13 years [38, 60–70]. The design for 26 studies was a community survey (cross-sectional) [22, 38, 41–43, 45–47, 50, 54–56, 58–69, 71], while it was a cross-sectional with simulated patient method for the remaining 13 studies [20, 21, 23–25, 39, 40, 44, 51–53, 57, 70]. The primary source of non-prescribed antibiotics access for children in 31 studies was retail drug outlets [20–25, 39–43, 45–58, 61, 62, 64, 65, 69, 70]. Antibiotic access sources for children in six of the remaining studies were mainly leftovers of home-stored drugs from previous prescriptions or uses [38, 60, 63, 66–68] and private clinics in the other two studies [44, 59]. Eighteen of the included studies were conducted in upper-middle-income countries [24, 25, 38, 39, 48, 49, 52, 58, 60–69], while the remaining twelve [21–23, 40, 44–47, 50, 51, 54, 57] and nine [20, 41–43, 53, 55, 56, 59, 70] studies were conducted in lower-middle-income and low-income countries, respectively. The children in 14 studies used non-prescribed antibiotics for acute diarrhea [20, 21, 23, 24, 39, 40, 43, 49–52, 54, 57, 70]; the children in nine studies used them for acute upper respiratory tract infections [25, 41, 42, 55, 59–63]; and the children in the remaining 16 studies used them for mixed-types of childhood illnesses [22, 38, 44–48, 53, 56, 58, 64–69]. Overall, all the individual studies included in our final analysis were from the LMICs. We found no eligible studies from the high-income countries since all of them were excluded during the screening and appraisal processes. Table 1 presents details of the characteristics of the studies included in this review.

**Table 1:** Characteristics of studies on community-level nonprescription antibiotic use for children, July 2022

Study	# of NP antibiotic use	Sample	Child age	Method	Major illness for which NP antibiotics used	Primary antibiotics source	Country	Income category [72]	Time of antibiotics use recall from the period of data collection
Abegaz et al. [20]	58	113	≤ 5 years	CS-SC	Acute diarrhea	Drug outlet	Ethiopia	Low-income	Instantly observed during data collection
Adeyemi et al. [54]	143	389	≤ 5 years	CS	Acute diarrhea	Drug outlet	Nigeria	Lower middle income	Within 2 months
Al-Noman and Elnimeiri [55]	354	581	≤ 5 years	CS	Acute RTI	Drug outlet	Yemen	Low income	Instantly observed during data collection
Al-Shawi et al. [60]	587	1030	≤ 12 years	CS	Acute RTI	Leftover	Saudi Arabia	Upper-middle-income	Ever recallable
Chang et al. [52]	143	256	≤ 7 years	CS-SC	Acute diarrhea	Drug outlet	China	Upper-middle-income	Instantly observed during data collection
Chang et al. [61]	1617	3358	5 years	CS	Acute cough	Drug outlet	China	Upper-middle-income	Within 6 months
Chang et al. [39]	1169	2411	≤ 5 years	CS-SC	Acute diarrhea	Drug outlet	China	Upper-middle-income	Instantly observed during data collection
Diwan et al. [21]	66	164	4 years	CS-SC	Acute diarrhea	Drug outlet	India	Lower-middle-income	Instantly observed during data collection
Edessa et al. [27]	67	100	≤ 13 years	CS-SC	Acute diarrhea	Drug outlet	Ethiopia	Low-income	Instantly observed during data collection
Hallit et al. [62]	79	202	≤ 12 years	CS	Acute RTI	Drug outlet	Lebanon	Upper-middle-income	Within 12 months
Hussain et al. [40]	258	355	5 years	CS-SC	Acute diarrhea	Drug outlet	Pakistan	Lower-middle-income	Instantly observed during data collection
Kibuule et al. [41]	86	199	≤ 5 years	CS	Acute RTI	Drug outlet	Uganda	Low-income	Within 1 month
Koji et al. [53]	166	262	≤ 2 years	CS-SC	Any illness	Drug outlet	Ethiopia	Low-income	Instantly observed during data collection
Lanyero et al. [42]	164	318	≤ 5 years	CS	Acute diarrhea	Drug outlet	Uganda	Low-income	Within 2 weeks
Lanyero et al. [43]	220	856	≤ 5 years	CS	Acute RTI	Drug outlet	Uganda	Low-income	Within 2 weeks
Lin et al. [62]	621	3579	≤ 13 years	CS	Any illness	Drug outlet	China	Upper-middle-income	Within 1 month
Lin et al. [63]	594	1465	≤ 13 years	CS	Acute RTI	Leftover	China	Upper-middle-income	Within 12 months
Malik et al. [44]	456	773	3–5 years	CS-SC	Acute RTI and Diarrhea	Clinic	Pakistan	Lower-middle-income	Instantly observed during data collection
Miyazaki et al. [22]	22	76	≤ 1 year	CS	Diarrhea, cough and fever	Drug outlet	Cambodia	Lower-middle-income	Within 2 weeks
Mukattash et al. [65]	332	855	≤ 12 years	CS	Fever and RTI	Drug outlet	Jordan	Upper-middle-income	Ever recallable
Nyeko et al. [56]	46	210	≤ 5 years	CS	Febrile illness	Drug outlet	Uganda	Low income	Within 2 weeks
Ocan et al. [59]	175	390	≤ 12 years	CS	Acute RTI	Clinic	Uganda	Low-income	Within 2 weeks
Ogbo et al. [23]	58	186	2.5 years	CS-SC	Acute diarrhea	Drug outlet	Nigeria	Lower-middle-income	Instantly observed during data collection
Paredes et al. [58]	120	231	≤ 5 years	CS	Any illness	Drug outlet	Peru	Upper-middle-income	Within 12 months
Saengcharoen et al. [24]	60	115	4 years	CS-SC	Acute diarrhea	Drug outlet	Thailand	Upper-middle-income	Instantly observed during data collection
Samir et al. [45]	478	2784	≤ 5 years	CS	Febrile Illness	Drug outlet	Bangladesh	Lower-middle-income	Within 2 weeks
Shet et al. [51]	92	146	4 years	CS-SC	Acute diarrhea	Drug outlet	India	Lower-middle-income	Instantly observed during data collection
Shi et al. [25]	58	73	4 years	CS-SC	Acute cough	Drug outlet	China	Upper-middle-income	Instantly observed during data collection
Simon et al. [46]	292	612	≤ 5 years	CS	Any illness	Drug outlet	Tanzania	Lower-middle-income	Within 12 months
Sun et al. [38]	4580	9838	≤ 13 years	CS	Any illness	Leftover	China	Upper-middle-income	Within 1 month
Togoobaatar et al. [47]	213	503	≤ 5 years	CS	Any illness	Drug outlet	Mongolia	Lower-middle-income	Within 6 months
Wu et al. [48]	172	1188	≤ 5 years	CS	Any illness	Drug outlet	China	Upper-middle-income	Within 6 months
Xu et al. [66]	410	1275	≤ 13 years	CS	Any illness	Leftover	China	Upper-middle-income	Within 1 month
Xu et al. [67]	402	1255	≤ 13 years	CS	Any illness	Leftover	China	Upper-middle-income	Within 1 month
Yu et al. [68]	529	854	≤ 12 years	CS	Any illness	Leftover	China	Upper-middle-income	Within 12 months
Yuan et al. [69]	330	1116	≤ 12 years	CS	Any illness	Drug outlet	China	Upper middle income	Within 12 months
Zawahir et al. [57]	135	316	5 years	CS-SC	Acute diarrhea	Drug outlet	Vietnam	Lower middle income	Instantly observed during data collection
Zhu et al. [71]	487	1211	≤ 5 years	CS	Acute diarrhea	Drug outlet	China	Upper-middle-income	Within 1 month
Zwisler et al. [50]	476	805	≤ 5 years	CS	Acute diarrhea	Drug outlet	India	Lower-middle-income	Within 2–3 days
Total	16,315	40,450							

^#^number, *CS* cross-sectional design, *NP* non-prescribed, *CS-SC* cross-sectional study with simulated case, *RTI* respiratory tract infection, and *WB* World Bank

### Risk of bias of the included studies

**Fig. 2:**
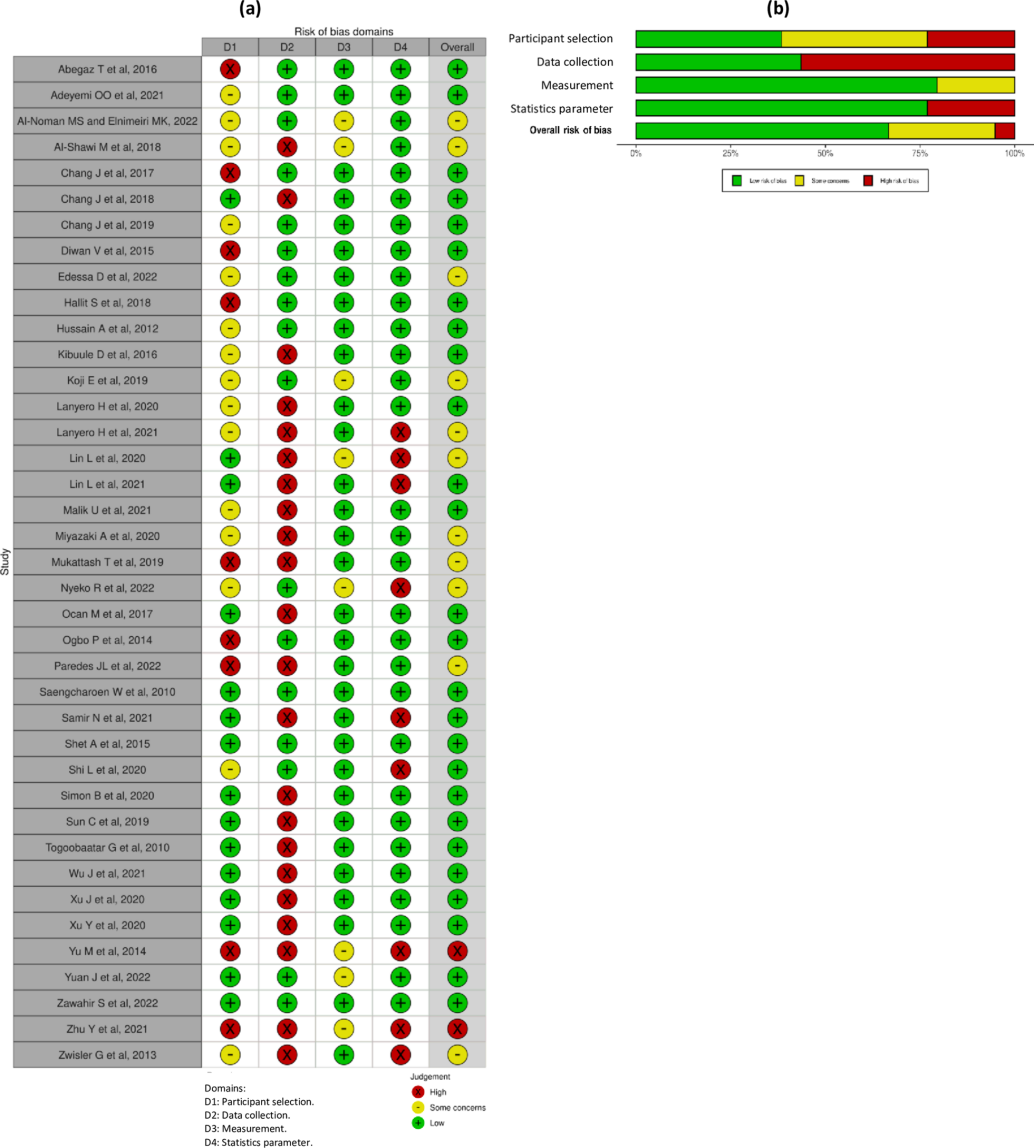
Risk of bias of the included individual studies. **a** Risk of bias summary for individual studies by domains. **b** Risk of bias graph by domains

The risk of bias was judged as of low level for the 26 studies we included [20, 21, 23–25, 38–42, 44–48, 51, 52, 54, 57, 59, 61–63, 66, 67, 69]. It was moderate (i.e., some concerns) and high levels in 11 [22, 43, 50, 53, 55, 56, 58, 60, 64, 65, 70] and two [49, 68] of the remaining studies, respectively. Most studies had a low or moderate risk of participant selection bias, while this was of a high level in nine studies [20, 21, 23, 49, 52, 58, 62, 65, 68]. Besides, 31 studies had a low level of risk for outcome measurement bias [20–25, 38–47, 50–52, 54, 57–59, 61–63, 65–67, 70], with some concerns in the remaining eight studies [53, 55, 56, 60, 64, 68, 69, 71]. However, 22 studies had a high risk of data collection bias [22, 38, 41–50, 58–61, 63–68]. With this, since the studies collected data on non-prescribed antibiotic use from caregivers/parents as a proxy for children, this technique might have led to the high risk of data collection bias based on the item considered in the tool. Figure 2 presents the risk of bias we assessed for the included studies. An additional file also shows the risk of bias appraised in more detail (see Additional file 3).

### Outcome measures

**Fig. 3:**
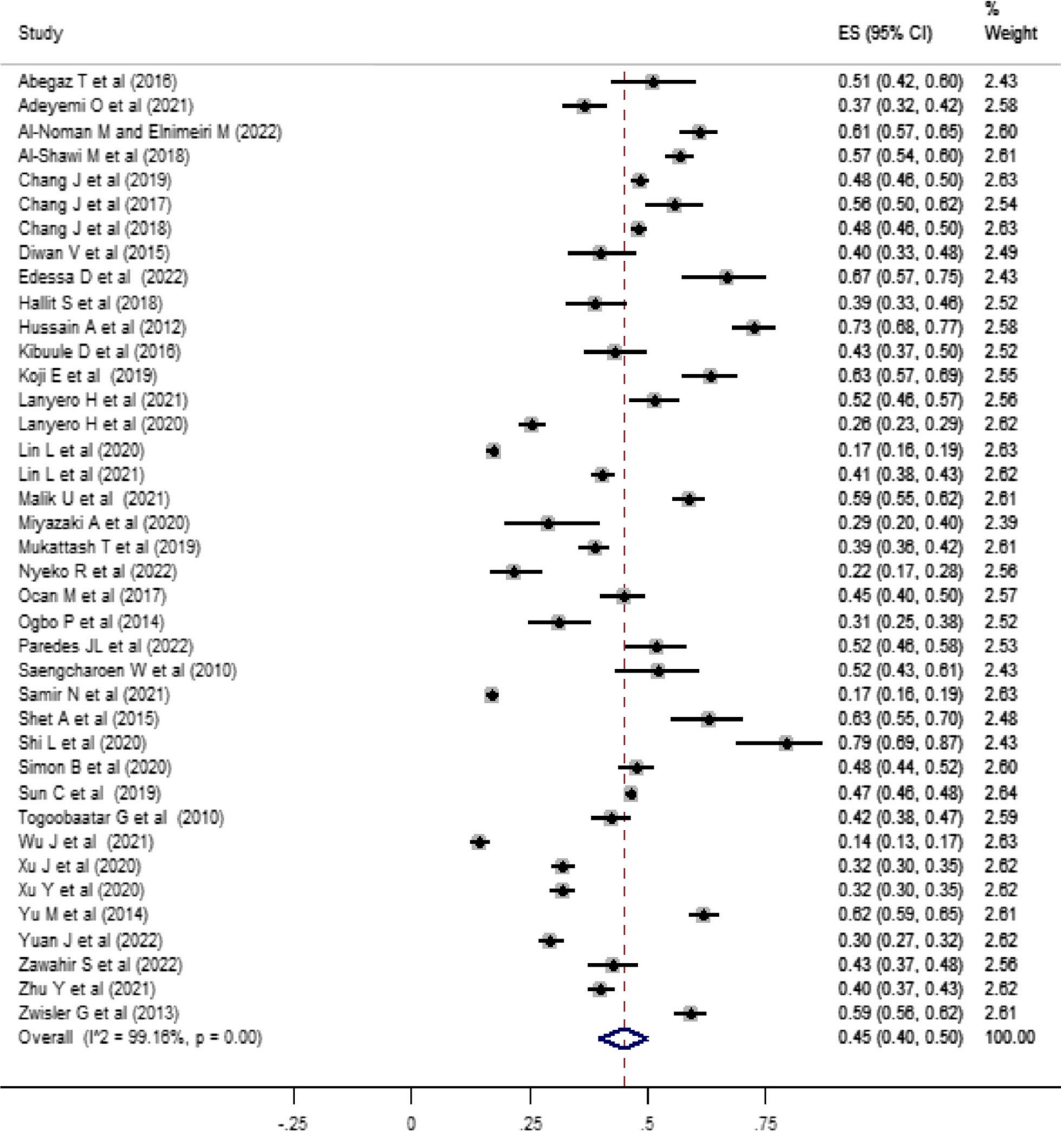
Forest plot for the proportion of community-level non-prescribed antibiotic use for children

The pooled estimate for the percentage of non-prescribed antibiotic use for children was 45% (95% CI: 40–50%; *I*^2^ = 99.16%; *P* < 0.001). The outcome measure for the pooled proportion is presented in Fig. 3. The percentage of antibiotics use for children without prescription in the individual studies range from 14% (95% CI: 13–17%) to 79% (95% CI: 69–87%).

#### Sensitivity and subgroup analyses

**Fig. 4:**
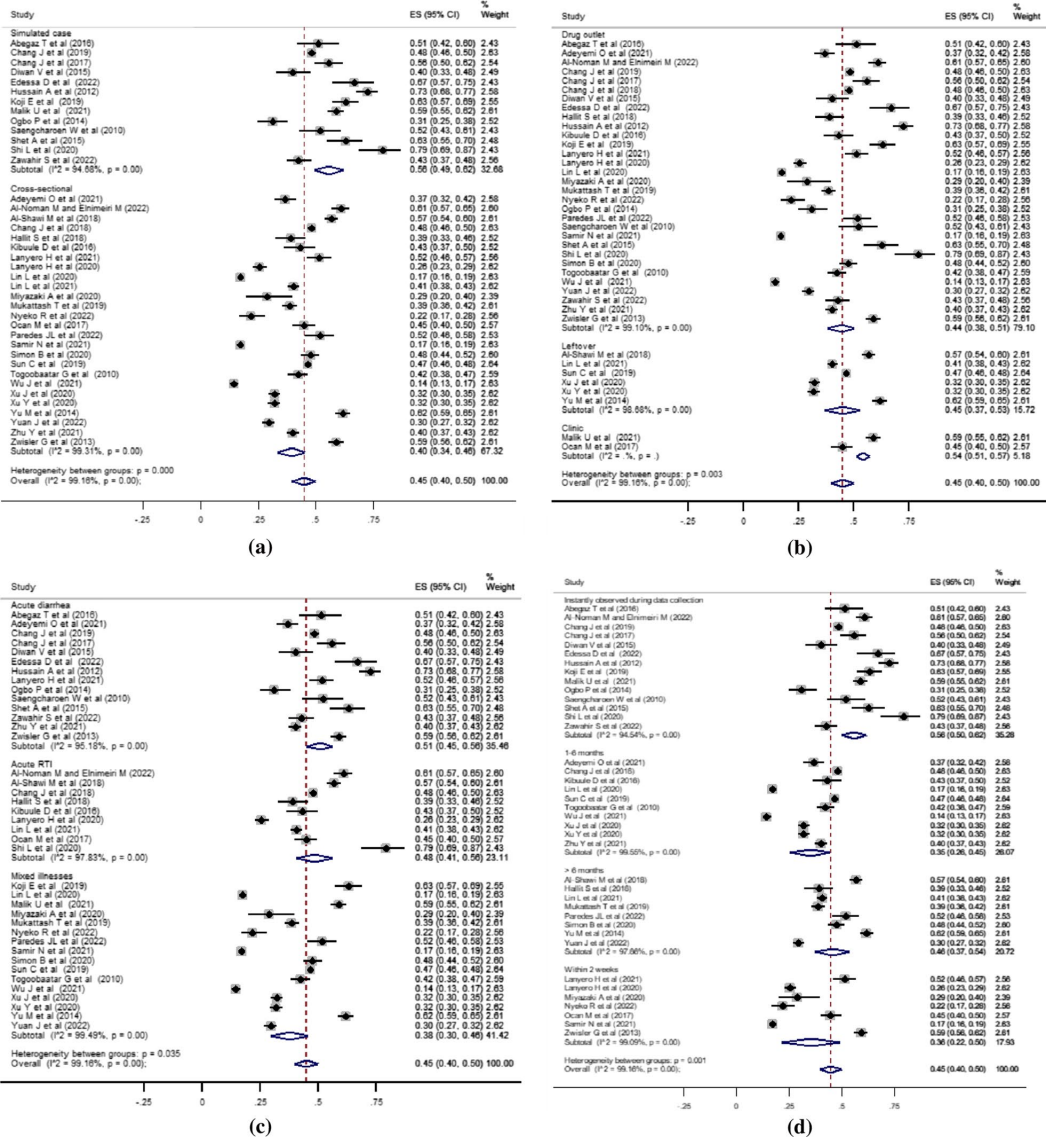
Forest plots of proportion on non-prescribed antibiotics use for children by subgroups. **a** Forest plot describing antibiotic uses by study methods. **b** Forest plot describing antibiotic uses by the primary antibiotic access sources. **c** Forest plot describing antibiotic uses by the common type of childhood illnesses. **d** Forest plot describing antibiotic uses by the time of data point from the data collection period of the studies

We performed several analyses to identify the source of heterogeneity among the combined studies. Initially, we conducted a sensitivity analysis by excluding outliers [25, 48] to see their effect on the degree of variability. However, the heterogeneity among the studies remained high (I^2^ = 99.08%). As a result, we included all the studies in the final meta-analysis model. Next, we carried out subgroup analyses based on the study method, primary sources of the non-prescribed antibiotics access, illness types for antibiotics indications, and the time of use recall reported or observed in the studies. Figure 4 presents details of the subgroup analyses by these parameters. Accordingly, the pooled estimate of the non-prescribed antibiotic use for children was 56% (95% CI: 49–62%; *I*^2^ = 94.68%; *P* < 0.001) for studies with simulated patient methods and 40% (95% CI: 34–46%; *I*^2^ = 99.31%; *P* < 0.001) for studies with cross-sectional survey. The degree of heterogeneity between groups for this subgrouping was statistically significant (*P* < 0.001). The estimate was 44% (95% CI: 38–51%; *I*^2^ = 99.10%; *P* < 0.001) for the primary non-prescribed antibiotics access from drug outlets, 45% (95% CI: 37–53%; *I*^2^ = 98.68%; *P* < 0.001) for the major accesses from home-stored leftovers, and 54% (95% CI: 51–57%; no calculated *I*^2^ and *P*-value) for accesses from clinics. The degree of heterogeneity between groups for this analysis was also statistically significant (*P* = 0.003). In addition, the pooled percentage estimates were 56% (95% CI: 50–62%; *I*^2^ = 94.54%; *P* < 0.001) for instantly observed antibiotics access at the time of data collection; 36% (95% CI: 22–50%; *I*^2^ = 99.09%; *P* < 0.001) for use recalls within two weeks; 35% (95% CI: 26–45%; *I*^2^ = 99.55%; *P* < 0.001) for use recalls between one and six months; and 46% (95% CI: 37–54%; *I*^2^ = 97.86%; *P* < 0.001) for use recalls longer than six months. The heterogeneity between groups was statistically significant (*P* = 0.001). Moreover, the subgroup analysis by the type of childhood illnesses for which the non-prescribed antibiotics were commonly sought showed a higher percentage estimate of 51% (95% CI: 45–56%; *I*^2^ = 95.18%; *P* < 0.001) for use in acute diarrhea than the estimates of 48% (95% CI: 41–56%; *I*^2^ = 97.83%; *P* < 0.001) for use in acute upper respiratory tract infections and 38% (95% CI: 30–46%; *I*^2^ = 99.49%; *P* < 0.001) for use in other mixed-illness types. The degree of heterogeneity between groups for this subgroup was also statistically significant (*P* = 0.035). However, disregarding the one study from Latin America [58], subgroup analysis by region of the study reports found no difference between the Asian and African countries. Besides, the subgroup analysis by the children’s age was also not significant. Additional files present findings of the subgroup analyses by the children’s age and region of the studies in more detail (see Additional files 4 and 5).

#### Publication bias

**Fig. 5:**
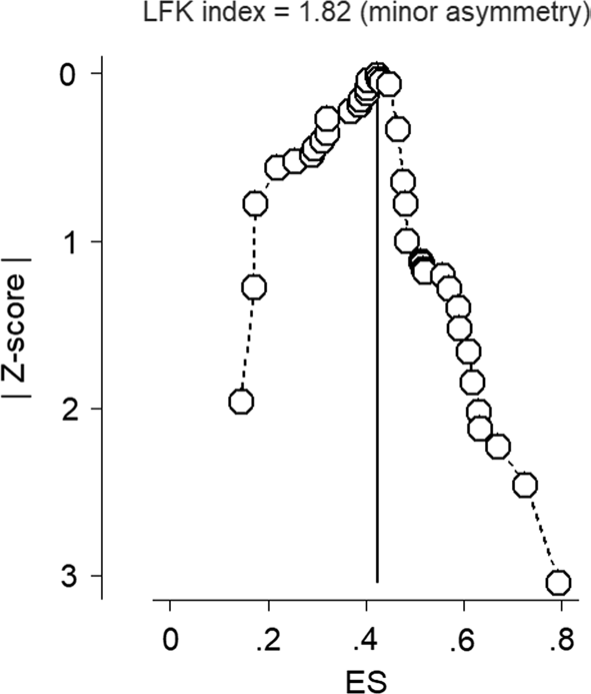
Doi plot of z-score value by effect sizes for publication bias

We initially performed an asymmetry check for the funnel plot to assess the presence of small-study effects (publication bias). The funnel plot appeared asymmetric and hinted to us about the availability of publication bias. Next, we tested the Luis Furuya-Kanamori (LFK) index using the Doi plot [37] alongside Egger’s regression *p*-value. The Egger’s regression test showed the presence of publication bias among the included studies (*P* = 0.033). The LFK index of 1.82 also revealed a minor asymmetry and suggests a low risk of publication bias. An additional file shows details of a funnel plot evaluated for publication bias (see Additional file 6). The heterogeneous population groups (i.e., children aged 0–13 years) and the different study designs (i.e., community survey, simulated patient methods, and small sample sizes) of the studies included in this review might have contributed to the asymmetry [73–75]. The LFK index could thus be recognized as a meaningful test since it valued the degree of asymmetry more than Egger’s regression test p-value. Egger’s p-value has no link with the degree of asymmetry. Figure 5 presents the publication bias assessed by using the LFK index.

## Discussion

In this study, we identified nearly half of the antibiotic uses for children in community settings were without prescriptions. The study settings for the entire studies included in this analysis were in the LMICs. The antibiotics use report without prescription was considerably higher for studies conducted using simulated patient methods than in studies conducted using a community survey. The retail drug outlets were the primary source of non-prescribed antibiotics access for children. Acute diarrhea, upper respiratory tract infections, and fever were the most common symptoms for which caregivers seek antibiotic treatments for their children without prescriptions.

Our estimate revealed that 45% of antibiotics used for children in the community settings were without prescriptions. The collective evidence from household-level antibiotic use studies in resource-limited countries also estimated a pooled percentage of 39% as nonprescription antibiotic uses [76]. A similar multicenter survey conducted in four LMICs also found the pediatric population as the most frequent user of antibiotics without prescriptions [77]. This mode of antibiotic access for children without prescriptions had links to managing common childhood illnesses [78]. In line with this, about two-thirds of acute childhood diarrhea and 80.5% of fever or cough cases led to the inappropriate receipt of antibiotics despite bacteria being not the common cause of acute non-bloody diarrhea and cough [18, 79]. In addition, the antibiotics have continued to be overused or misused for treatments of fever, cough, acute upper respiratory tract infections, and non-bloody diarrhea [2, 80–82]. The primary driver for these practices of antibiotic overuse is patient demand [83]. Several other studies from different settings in the LMICs also reported similar percentages of non-prescribed antibiotic use by communities as the usual unnecessary or inappropriate options for most self-limiting illnesses. This unsafe antibiotic practice spanned from 36.1% to 73.2% [84–100]. The non-prescribed antibiotic use for children was relatively higher among under-five children (i.e., 46%) than in the subgroup of children under 13 years (i.e., 41%). Similarly, a systematic analysis of survey data from LMICs revealed the increasing trends in antibiotic use for common self-limiting illnesses such as fever, diarrhea, and cough in under-five children [101]. There was also a high rate of home treatment for the perceived pneumonia in under-five children, particularly with antibiotics [102].

The high threshold of population-level antibiotic use for children without prescriptions increases the chance of resistance [103]. The widespread and inappropriate antibiotic use are the primary driving forces behind antibiotic resistance [104, 105]. Even in the presence of legitimate prescription for antibiotics, random discontinuation of these medicines after relief from illness symptoms and their subsequent storage as leftovers at home for later uses have become the other habitual unsafe practices with the antibiotics [106]. Other studies also emphasized incomplete antibiotic courses that emanate from the treatment non-compliance as the most common risk factor for resistance development [107, 108]. A lack of parental awareness about antibiotic resistance might superimpose the incomplete antibiotic usage by the children [109]. In our finding, this unsafe antibiotic practice for children uses at the community levels was confined to parents or caregivers from the LMICs despite the non-restrictive searches of evidence we conducted. There are practice and culture-specific aspects of uncertainty avoidance that can explain the cross-country variability in their policies and regulations of antibiotics utilization [110, 111]. In this regard, unlike the LMICs, some high-income countries have developed pediatric antibiotic stewardship programs, networks, and guidelines [112]. Again, evidence from some studies revealed a positive relationship between socioeconomic marginalization and the increased burden of childhood morbidities [113, 114]. Such socioeconomic inequality is among the predictors of variability for common childhood morbidities [115]. Besides, low immunization coverage in the poorer settings can explain their higher burden of pediatric infections than in the richer ones [115–117]. About 90% of the global estimate for vaccine-preventable rotavirus disease-associated deaths in under-five children occurred in Asia and sub-Saharan Africa, with ten countries contributing to most deaths [118]. Other contributors to acute respiratory tract infections and diarrheal morbidities in children of the LMICs have been unimproved water, sanitation, and hygiene facilities [119]. In these less-developed countries, patients often overuse and misuse antibiotics to manage illnesses they frequently encounter [120]. Indeed, there have been clear epidemiological transitions related to causes of childhood morbidity and mortality in some upper-middle-income countries [121]. However, about half of the studies that report non-prescribed antibiotic use for children were from these countries (see Table 1). It marks a habitual continuation of this risky antibiotic practice.

Essentially, up to 93% of children in the LMICs obtain the usual care for childhood illnesses using antibiotics without prescriptions [122]. Awareness gaps concerning antibiotic resistance had explained about half (i.e., 47.7%) of the antibiotic use for children [123], and this is clear from the usual antibiotic-sharing habits of the community [124]. The worst scenario with antibiotics use without prescription can involve the watch group antibiotics (i.e., the antibiotics classes with a high risk of resistance selection for which they obtained a priority of limited use for some specific infections with careful monitoring) [19]. The use of this antibiotic class without correct indication appears to be an increasing habit in the contemporary world. A study in Vietnam also confirms this trend in which 54.3% of the antibiotic encounters for children use were the watch class antibiotics [12]. Although there are several public health problems related to non-prescribed antibiotics use at all levels and settings around the globe [125], responses of all concerned bodies in the LMICs appeared very minimal and underscored.

The estimate of non-prescribed antibiotic use for children was inconsistent by measurement methods, time of use recall, and the illness types for which the antibiotics were sought [84]. Our estimate showed a significantly higher non-prescribed antibiotic use for children in simulated patient methods (ranges from 49 to 62%) than in the community-based use history survey (ranges from 34 to 46%) (*P* < 0.001) [126, 127]. Despite a likely bias with the simulated conversation, it has been a domain of the recent approaches that can successfully identify the actual antibiotic practice of drug retailers, with the capacity to reduce the possible underreporting inherent with cross-sectional survey designs [127, 128]. Integrating observed encounters with surveys can meaningfully account for providers’ actual antibiotic dispensing practices since simulations may introduce bias and can miss some sources of non-prescribed antibiotics [6]. From our analysis, the retail drug outlets were the most common sources of access for non-prescribed antibiotic uses [129, 130]. In this regard, retail drug outlets were the sources for about two-thirds (62%) of the global nonprescription antibiotic use [131]. Non-official and home-stored leftover accesses are also potential sources of unsafe antibiotic practices [132]. Besides, drug outlets and non-official suppliers were the primary enablers of unrestricted access to antibiotics, including broad spectrums that could have a high chance of misuse and resistance selection [133]. Home storages of prescribed antibiotics with the intent for later uses are other modes of misuse for the previous indication (i.e., immature discontinuation of the dosage course) and the current intention (i.e., inappropriate dose and activity for the intended illness is unknown) [134]. A common implication of unrestricted antibiotic access involved aspects of awareness gaps among providers and users about the risk of antibiotic resistance in resource-limited settings [135, 136].

## Limitations

Although we considered searches of worldwide data from common databases to retrieve relevant records, this systematic analysis has some limitations. First, the considered study settings have covered a wide range of global contexts that are highly variable for different reasons. Inconsistent sampling frames are expected and can contribute to cultural, clinical, and methodological heterogeneity of antibiotic uses and regulations. However, in our analysis, we considered the random-effects method of the meta-analysis as an assumption to account for the different effect sizes that possibly emanate from the diverse sampling frames [33, 34]. In addition, we employed subgroup analyses to identify and recognize the sources of this heterogeneity across studies [33]. Second, our data screening and eligibility assessment process also excluded studies published in languages other than English. This process might have underestimated the non-prescribed antibiotic uses for children at the community levels [137]. In this regard, studies that compared the impacts of restricting evidence syntheses to English-language publications with the analyses adding non-English languages reported little difference in the effect estimates and conclusions of the systematic analyses. However, these studies were non-conclusive since they suggest comprehensive searches and further studies [138–141]. Finally, the included studies report non-prescribed antibiotic uses at varying recall times. This approach might have introduced a recall bias for studies reporting longer months of use history assessed in the community surveys. Therefore, the interpretations of the findings in this systematic review and meta-analysis should consider these limitations.

## Conclusion

We found that community-level non-prescribed antibiotic use for children by caregivers in the low- and middle-income countries accounted for about half of the antibiotics accessed in these settings. The report on this risky practice was mainly from East Asia, South Asia, and sub-Saharan African countries. The drug outlets were the primary access points for the antibiotics, while unofficial and home-stored leftovers were also the other sources of unrestricted access. Fever, acute diarrhea, and upper respiratory tract infections were the most frequent childhood illness types for antibiotics use without prescriptions. This unsafe antibiotic practice for children’s use without a prescription is a high threshold for community-level use and is a threatening issue to global public health. Therefore, we recommend a context-specific educational and regulatory interventions at these outlets and the community levels, targeting responsibility gaps of providers, caregivers, and regulatory bodies as the first step to improving antibiotic usage for children in low- and middle-income countries.

## Supplementary Information

**Additional file 1. **Completed PRISMA checklist.

**Additional file 2.** Methodological quality assessment of individual studies.

**Additional file 3.** Risk of bias assessed for the included individual studies.

**Additional file 4. **Nonprescription antibiotic use for children by region.

**Additional file 5. **Nonprescription antibiotic use for children by the children’s age.

**Additional file 6. **Funnel plot for publication bias.

## Data Availability

All relevant data are within the manuscript and its additional files.

## References

[1] O'Neill J: Tackling drug-resistant infections globally: final report and recommendations. 2016, https://amr-review.org/sites/default/files/160525_Final%20paper_with%20cover.pdf. Accessed February 27, 2022.

[2] Auta A, Hadi MA, Oga E, Adewuyi EO, Abdu-Aguye SN, Adeloye D, Strickland-Hodge B, Morgan DJ (2019). Global access to antibiotics without prescription in community pharmacies: a systematic review and meta-analysis. J Infect.

[3] Grigoryan L, Germanos G, Zoorob R, Juneja S, Raphael JL, Paasche-Orlow MK, Trautner BW (2019). Use of antibiotics without a prescription in the U.S. population: a scoping review. Ann Intern Med.

[4] Belachew SA, Hall L, Selvey LA (2021). Non-prescription dispensing of antibiotic agents among community drug retail outlets in Sub-Saharan African countries: a systematic review and meta-analysis. Antimicrob Resist Infect Control.

[5] Torres NF, Chibi B, Kuupiel D, Solomon VP, Mashamba-Thompson TP, Middleton LE (2021). The use of non-prescribed antibiotics; prevalence estimates in low-and-middle-income countries. A systematic review and meta-analysis. Arch Public Health.

[6] Padget M, Guillemot D, Delarocque-Astagneau E (2016). Measuring antibiotic consumption in low-income countries: a systematic review and integrative approach. Int J Antimicrob Agents.

[7] Collignon P, Beggs JJ, Walsh TR, Gandra S, Laxminarayan R (2018). Anthropological and socioeconomic factors contributing to global antimicrobial resistance: a univariate and multivariable analysis. Lancet Planet Health.

[8] Ocan M, Obuku EA, Bwanga F, Akena D, Richard S, Ogwal-Okeng J, Obua C (2015). Household antimicrobial self-medication: a systematic review and meta-analysis of the burden, risk factors and outcomes in developing countries. BMC Public Health.

[9] Torres NF, Chibi B, Middleton LE, Solomon VP, Mashamba-Thompson TP (2019). Evidence of factors influencing self-medication with antibiotics in low and middle-income countries: a systematic scoping review. Public Health.

[10] Zoorob R, Grigoryan L, Nash S, Trautner BW (2016). Nonprescription antimicrobial use in a primary care population in the United States. Antimicrob Agents Chemother.

[11] Ivanovska V, Zdravkovska M, Bosevska G, Angelovska B (2013). Antibiotics for upper respiratory infections: public knowledge, beliefs and self-medication in the Republic of Macedonia. Prilozi.

[12] Nguyen NV, Do NTT, Nguyen CTK, Tran TK, Ho PD, Nguyen HH, Vu HTL, Wertheim HFL, van Doorn HR, Lewycka S (2020). Community-level consumption of antibiotics according to the AWaRe (Access, Watch, Reserve) classification in rural Vietnam. JAC Antimicrob Resist.

[13] Chalker J, Ratanawijitrasin S, Chuc NT, Petzold M, Tomson G (2005). Effectiveness of a multi-component intervention on dispensing practices at private pharmacies in Vietnam and Thailand–a randomized controlled trial. Soc Sci Med.

[14] Blanchard J, Solaipandian M, John EB, Pandith M, Jeo B, Saji S, Kumar A, May L, Davey K, Douglass K (2021). Self-prescribing of antibiotics by patients seeking care in Indian emergency departments. J Am Coll Emerg Physicians Open.

[15] Afari-Asiedu S, Hulscher M, Abdulai MA, Boamah-Kaali E, Asante KP, Wertheim HFL (2020). Every medicine is medicine; exploring inappropriate antibiotic use at the community level in rural Ghana. BMC Public Health.

[16] Nguyen HH, Ho DP, Vu TLH, Tran KT, Tran TD, Nguyen TKC, van Doorn HR, Nadjm B, Kinsman J, Wertheim H (2019). "I can make more from selling medicine when breaking the rules" - understanding the antibiotic supply network in a rural community in Viet Nam. BMC Public Health.

[17] Batista AD, Figueiras A, Zapata-Cachafeiro M, Roque F, Herdeiro MT (2020). Antibiotic dispensation without a prescription worldwide: a systematic review. Antibiotics.

[18] Fink G, D'Acremont V, Leslie HH, Cohen J (2020). Antibiotic exposure among children younger than 5 years in low-income and middle-income countries: a cross-sectional study of nationally representative facility-based and household-based surveys. Lancet Infect Dis.

[19] WHO: The 2019 WHO AWaRe classification of antibiotics for evaluation and monitoring of use. In: The 2019 WHO AWaRe classification of antibiotics for evaluation and monitoring of use. edn. https://www.who.int/news/item/01-10-2019-who-releases-the-2019-aware-classification-antibiotics. Accessed February 27, 2022.

[20] Abegaz TM, Belachew SA, Abebe TB, Gebresilassie BM, Teni FS, Woldie HG (2016). Management of children’s acute diarrhea by community pharmacies in five towns of Ethiopia: simulated client case study. Ther Clin Risk Manag.

[21] Diwan V, Sabde YD, Byström E, De Costa A (2015). Treatment of pediatric diarrhea: a simulated client study at private pharmacies of Ujjain, Madhya Pradesh, India. J Infect Dev Ctries.

[22] Miyazaki A, Tung R, Taing B, Matsui M, Iwamoto A, Cox SE (2020). Frequent unregulated use of antibiotics in rural Cambodian infants. Trans R Soc Trop Med Hyg.

[23] Ogbo PU, Aina BA, Aderemi-Williams RI (2014). Management of acute diarrhea in children by community pharmacists in Lagos, Nigeria. Pharm Pract.

[24] Saengcharoen W, Lerkiatbundit S (2010). Practice and attitudes regarding the management of childhood diarrhoea among pharmacies in Thailand. Int J Pharm Pract.

[25] Shi L, Chang J, Liu X, Zhai P, Hu S, Li P, Hayat K, Kabba JA, Feng Z, Yang C (2020). Dispensing antibiotics without a prescription for acute cough associated with common cold at community pharmacies in shenyang, northeastern china: a cross-sectional study. Antibiotics.

[26] Page MJ, McKenzie JE, Bossuyt PM, Boutron I, Hoffmann TC, Mulrow CD, Shamseer L, Tetzlaff JM, Akl EA, Brennan SE (2021). The PRISMA 2020 statement: an updated guideline for reporting systematic reviews. BMJ.

[27] Edessa D, Oljira L, Dinsa GD, Asefa F, Assefa N, Dessie Y: Community-level nonprescription and leftover antibiotic uses for children in low and middle-income countries: a meta-analysis of observational studies. 2021, https://www.crd.york.ac.uk/prospero/display_record.php?ID=CRD4202128897. Accessed 24 Feb 2022.

[28] Hoy D, Brooks P, Woolf A, Blyth F, March L, Bain C, Baker P, Smith E, Buchbinder R (2012). Assessing risk of bias in prevalence studies: modification of an existing tool and evidence of interrater agreement. J Clin Epidemiol.

[29] Migliavaca CB, Stein C, Colpani V, Munn Z, Falavigna M (2020). Quality assessment of prevalence studies: a systematic review. J Clin Epidemiol.

[30] McGuinness LA, Higgins JPT (2021). Risk-of-bias VISualization (robvis): an R package and Shiny web app for visualizing risk-of-bias assessments. Res Synth Methods.

[31] Ribaldone DG, Petrini E (2017). How to interpret meta-analysis results. Panminerva Med.

[32] Higgins J, Thompson S, Deeks J, Altman D (2002). Statistical heterogeneity in systematic reviews of clinical trials: a critical appraisal of guidelines and practice. J Health Serv Res Policy.

[33] Richardson M, Garner P, Donegan S (2019). Interpretation of subgroup analyses in systematic reviews: a tutorial. Clin Epidemiol Glob Health.

[34] Borenstein M, Higgins J (2013). Meta-analysis and subgroups. Prev Sci.

[35] Ioannidis JP, Trikalinos TA (2007). The appropriateness of asymmetry tests for publication bias in meta-analyses: a large survey. CMAJ.

[36] Boutron I, Page MJ, Higgins JP, Altman DG, Lundh A, Hróbjartsson A. Considering bias and conflicts of interest among the included studies. In: Cochrane Handbook for Systematic Reviews of Interventions. Wiley Online Library. 2019;177-204. 10.1002/9781119536604.ch7.

[37] Furuya-Kanamori L, Barendregt JJ, Doi SAR (2018). A new improved graphical and quantitative method for detecting bias in meta-analysis. Int J Evid Based Healthc.

[38] Sun C, Hu YJ, Wang X, Lu J, Lin L, Zhou X (2019). Influence of leftover antibiotics on self-medication with antibiotics for children: a cross-sectional study from three Chinese provinces. BMJ Open.

[39] Chang J, Xu S, Zhu S, Li Z, Yu J, Zhang Y, Zu J, Fang Y, Ross-Degnan D (2019). Assessment of non-prescription antibiotic dispensing at community pharmacies in China with simulated clients: a mixed cross-sectional and longitudinal study. Lancet Infect Dis.

[40] Hussain A, Ibrahim MI (2012). Management of diarrhoea cases by community pharmacies in 3 cities of Pakistan. EMHJ.

[41] Kibuule D, Kagoya HR, Godman B (2016). Antibiotic use in acute respiratory infections in under-fives in Uganda: findings and implications. Expert Rev Anti Infect Ther.

[42] Lanyero H, Eriksen J, Obua C, StålsbyLundborg C, Nanzigu S, Katureebe A, Kalyango JN, Ocan M (2020). Use of antibacterials in the management of symptoms of acute respiratory tract infections among children under five years in Gulu, northern Uganda: prevalence and determinants. PLoS ONE.

[43] Lanyero H, Ocan M, Obua C, StålsbyLundborg C, Nanzigu S, Katureebe A (2021). J NK, Eriksen J: Antibiotic use among children under five years with diarrhea in rural communities of Gulu, northern Uganda: a cross-sectional study. BMC Public Health.

[44] Malik UR, Chang J, Hashmi F, Atif N, Basir H, Hayat K, Khan FU, Kabba JA, Lambojon K, Fang Y (2021). A simulated client exploration of nonprescription dispensing of antibiotics at drugstores for pediatric acute diarrhea and upper respiratory infection in Lahore, Pakistan. Infect Drug Resist.

[45] Samir N, Hassan MZ, Biswas MAAJ, Chowdhury F, Akhtar Z, Lingam R, Banu S, Homaira N (2021). Antibiotic use for febrile illness among under-5 children in Bangladesh: a nationally representative sample survey. Antibiotics.

[46] Simon B, Kazaura M (2020). Prevalence and factors associated with parents self-medicating under-fives with antibiotics in bagamoyo district council, tanzania: a cross-sectional study. Patient Prefer Adherence.

[47] Togoobaatar G, Ikeda N, Ali M, Sonomjamts M, Dashdemberel S, Mori R, Shibuya K (2010). Survey of non-prescribed use of antibiotics for children in an urban community in Mongolia. Bull World Health Organ.

[48] Wu J, Yang F, Yang H, Zhang G, Mu K, Feng J, Wang J, Yin X (2021). Prevalence of antibiotic self-medication behavior and related factors among children aged 0 to 5 years. Expert Rev Anti Infect Ther.

[49] Zhu Y, Luo S, He H, Yan R, Zhou Y, Deng X, Tang X (2020). Non-prescription antibiotic use for cough among chinese children aged under 5 years: a community-based cross-sectional study. BMJ Open.

[50] Zwisler G, Simpson E, Moodley M (2013). Treatment of diarrhea in young children: results from surveys on the perception and use of oral rehydration solutions, antibiotics, and other therapies in India and Kenya. J Glob Health.

[51] Shet A, Sundaresan S, Forsberg BC (2015). Pharmacy-based dispensing of antimicrobial agents without prescription in India: appropriateness and cost burden in the private sector. Antimicrob Resist Infect Control.

[52] Chang J, Ye D, Lv B, Jiang M, Zhu S, Yan K, Tian Y, Fang Y (2017). Sale of antibiotics without a prescription at community pharmacies in urban China: a multicentre cross-sectional survey. J Antimicrob Chemother.

[53] Koji EM, Gebretekle GB, Tekle TA (2019). Practice of over-the-counter dispensary of antibiotics for childhood illnesses in Addis Ababa, Ethiopia: a simulated patient encounter study. Antimicrob Resist Infect Control.

[54] Adeyemi OO, Alabi AS, Adeyemi OA, Talabi OT, Abidakun OM, Joel IY, Stonehouse NJ (2021). Acute gastroenteritis and the usage pattern of antibiotics and traditional herbal medications for its management in a Nigerian community. PLoS ONE.

[55] Al-Noban MS, Elnimeiri MK (2022). Mothers knowledge, attitude and practices regarding acute respiratory infection in children under five years/urban and rural Areas-Al Mukalla city-2022. EJUA-BA.

[56] Nyeko R, Otim F, Obiya EM, Abala C (2022). Pre-hospital exposures to antibiotics among children presenting with fever in northern Uganda: a facility-based cross-sectional study. BMC Pediatr.

[57] Zawahir S, Le HTT, Nguyen T-A, Beardsley J, Dang AD, Bernays S, Viney K, Cao TH, Drabarek D, Tran HH (2022). Inappropriate supply of antibiotics for common viral infections by community pharmacies in Vietnam: a standardised patient survey. Lancet Reg Health West Pac.

[58] Paredes JL, Navarro R, Watanabe T, Morán F, Balmaceda MP, Reateguí A, Elias R, Bardellini M, Ochoa TJ (2022). Knowledge, attitudes and practices of parents towards antibiotic use in rural communities in Peru: a cross-sectional multicentre study. BMC Public Health.

[59] Ocan M, Aono M, Bukirwa C, Luyinda E, Ochwo C, Nsambu E, Namugonza S, Makoba J, Kandaruku E, Muyende H (2017). Medicine use practices in management of symptoms of acute upper respiratory tract infections in children (≤12 years) in Kampala city, Uganda. BMC Public Health.

[60] Al-Shawi MM, Darwish MA, Wahab MMA, Al-Shamlan NA (2018). Misconceptions of parents about antibiotic use in upper respiratory tract infections: A survey in primary schools of the Eastern province, KSA. J Fam Community Med.

[61] Chang J, Lv B, Zhu S, Yu J, Zhang Y, Ye D, Aziz MM, Yang C, Fang Y (2018). Non-prescription use of antibiotics among children in urban China: a cross-sectional survey of knowledge, attitudes, and practices. Expert Rev Anti Infect Ther.

[62] Hallit S, Zahreddine L, Saleh N, Shakaroun S, Lahoud N (2020). Practice of parents and pharmacists regarding antibiotics use in pediatrics: A 2017 cross-sectional study in Lebanese community pharmacies. J Eval Clin Pract.

[63] Lin L, Harbarth S, Hargreaves JR, Zhou X, Li L (2021). Large-scale survey of parental antibiotic use for paediatric upper respiratory tract infections in China: implications for stewardship programmes and national policy. Int J Antimicrob Agents.

[64] Lin L, Harbarth S, Wang X, Zhou X (2020). Survey of parental use of antimicrobial drugs for common childhood infections, China. Emerg Infect Dis.

[65] Mukattash TL, Alkhatatbeh MJ, Andrawos S, Jarab AS, AbuFarha RK, Nusair MB (2020). Parental self-medication of antibiotics for children in Jordan. J Pharm Health Serv Res.

[66] Xu J, Wang X, Sun KS, Lin L, Zhou X (2020). Parental self-medication with antibiotics for children promotes antibiotic over-prescribing in clinical settings in China. Antimicrob Resist Infect Control.

[67] Xu Y, Lu J, Sun C, Wang X, Hu YJ, Zhou X (2020). A cross-sectional study of antibiotic misuse among Chinese children in developed and less developed provinces. J Infect Dev Ctries.

[68] Yu M, Zhao G, StålsbyLundborg C, Zhu Y, Zhao Q, Xu B (2014). Knowledge, attitudes, and practices of parents in rural China on the use of antibiotics in children: a cross-sectional study. BMC Infect Dis.

[69] Yuan J, Du W, Li Z, Deng Q, Ma G (2022). Prevalence and risk factors of self-medication among the pediatric population in China: a national survey. Front Public Health.

[70] Edessa D, Sisay M, Hagos B, Amare F (2022). Antimicrobial use and management of childhood diarrhea at community drug retail outlets in eastern Ethiopia: a matched questionnaire-based and simulated patient-case study. Pediatric Health Med Ther.

[71] Zhu Y, Tang X, Yan R, Shao Z, Zhou Y, Deng X, Luo S, He H (2021). Non-prescription antibiotic use for cough among Chinese children under 5 years of age: a community-based cross-sectional study. BMJ Open.

[72] WB: World Bank Country and Lending Groups: Country Classification. 2021, https://datahelpdesk.worldbank.org/knowledgebase/articles/906519-world-bank-country-and-lending-groups. Accessed 25 Feb 2022.

[73] Sedgwick P, Marston L (2015). How to read a funnel plot in a meta-analysis. BMJ.

[74] Sterne JA, Sutton AJ, Ioannidis JP, Terrin N, Jones DR, Lau J, Carpenter J, Rücker G, Harbord RM, Schmid CH (2011). Recommendations for examining and interpreting funnel plot asymmetry in meta-analyses of randomised controlled trials. BMJ.

[75] Drucker AM, Fleming P, Chan AW (2016). Research techniques made simple: assessing risk of bias in systematic reviews. J Invest Dermatol.

[76] Ocan M, Obuku EA, Bwanga F, Akena D, Richard S, Ogwal-Okeng J, Obua C (2015). Household antimicrobial self-medication: a systematic review and meta-analysis of the burden, risk factors and outcomes in developing countries. BMC Public Health.

[77] Ingelbeen B, Koirala KD, Verdonck K, Barbé B, Mukendi D, Thong P, El Safi S, Van Duffel L, Bottieau E, van der Sande MAB (2021). Antibiotic use prior to seeking medical care in patients with persistent fever: a cross-sectional study in four low- and middle-income countries. Clin Microbiol Infect.

[78] Aslam A, Gajdács M, Zin CS, Ab Rahman NS, Ahmed SI, Zafar MZ, Jamshed S (2020). Evidence of the practice of self-medication with antibiotics among the lay public in low-and middle-income countries: a scoping review. Antibiotics.

[79] Tsige AG, Nedi T, Bacha T (2020). Assessment of the management of diarrhoea among children under five in Addis Ababa, Ethiopia. Pediatric Health Med Ther.

[80] Thobari JA, Satria CD, Ridora Y, Watts E, Handley A, Samad S, Bachtiar NS, Bines JE, Soenarto Y, Buttery JP (2019). Antimicrobial use in an Indonesian community cohort 0–18 months of age. PLoS ONE.

[81] Deng P, Yu J, Zhou N, Hu M (2018). Access to medicines for acute illness and antibiotic use in residents: a medicines household survey in Sichuan Province, western China. PLoS ONE.

[82] Erku DA, Mekuria AB, Belachew SA (2017). Inappropriate use of antibiotics among communities of Gondar town, Ethiopia: a threat to the development of antimicrobial resistance. Antimicrob Resist Infect Control.

[83] Tillekeratne LG, Bodinayake CK, Dabrera T, Nagahawatte A, Arachchi WK, Sooriyaarachchi A, Stewart K, Watt M, Østbye T, Woods CW (2017). Antibiotic overuse for acute respiratory tract infections in Sri Lanka: a qualitative study of outpatients and their physicians. BMC Fam Pract.

[84] Ateshim Y, Bereket B, Major F, Emun Y, Woldai B, Pasha I, Habte E, Russom M (2019). Prevalence of self-medication with antibiotics and associated factors in the community of Asmara, Eritrea: A descriptive cross sectional survey. BMC Public Health.

[85] Belachew SA, Hall L, Selvey LA (2021). Non-prescription dispensing of antibiotic agents among community drug retail outlets in Sub-Saharan African countries: a systematic review and meta-analysis. Antimicrob Resist Infect Control.

[86] Bogale AA, Amhare AF, Chang J, Bogale HA, Betaw ST, Gebrehiwot NT, Fang Y (2019). Knowledge, attitude, and practice of self-medication with antibiotics among community residents in Addis Ababa, Ethiopia. Expert Rev Anti Infect Ther.

[87] Do NTT, Vu HTL, Nguyen CTK, Punpuing S, Khan WA, Gyapong M, Asante KP, Munguambe K, Gómez-Olivé FX, John-Langba J (2021). Community-based antibiotic access and use in six low-income and middle-income countries: a mixed-method approach. Lancet Glob Health.

[88] ElongEkambi GA, OkallaEbongue C, Penda IC, Nnanga Nga E, Mpondo Mpondo E, EboumbouMoukoko CE (2019). Knowledge, practices and attitudes on antibiotics use in Cameroon: Self-medication and prescription survey among children, adolescents and adults in private pharmacies. PLoS ONE.

[89] Poyongo BP, Sangeda RZ (2020). Pharmacists’ knowledge, attitude and practice regarding the dispensing of antibiotics without prescription in tanzania: an explorative cross-sectional study. Pharmacy.

[90] Haque M, Rahman NAA, McKimm J, Kibria GM, Majumder MAA, Haque SZ, Islam MZ, Abdullah SLB, Daher AM, Zulkifli Z (2019). Self-medication of antibiotics: investigating practice among university students at the Malaysian national defence university. Infect Drug Resist.

[91] Karuniawati H, Hassali MAA, Suryawati S, Ismail WI, Taufik T, Hossain MS (2021). Assessment of knowledge, attitude, and practice of antibiotic use among the population of boyolali, indonesia: a cross-sectional study. Int J Environ Res Public Health.

[92] Kuang L, Liu Y, Wei W, Song X, Li X, Liu Q, Hong W, Liu Q, Li J, Chen Z (2020). Non-prescription sale of antibiotics and service quality in community pharmacies in Guangzhou, China: a simulated client method. PLoS ONE.

[93] Nusair MB, Al-azzam S, Alhamad H, Momani MY (2021). The prevalence and patterns of self-medication with antibiotics in Jordan: a community-based study. Int J Clin Pract.

[94] Srour K. Self-medication and over-the-counter dispensing of antibiotics in Egypt_ prevalence, reasons and outcomes. A systematic review. UiT The Arctic University of Norway. 2020. https://hdl.handle.net/10037/20260.

[95] Hammour KA, Jalil MA, Hammour WA (2018). An exploration of parents' knowledge, attitudes and practices towards the use of antibiotics in childhood upper respiratory tract infections in a tertiary Jordanian Hospital. SPJ.

[96] Nasimfar A, Sadeghi E, AmuzMehr A (2018). Evaluation of knowledge, attitude, and practice of parents on the use of antibiotics for acute upper respiratory tract infections in children admitted to Motahari Hospital of Urmia in 2017–2018. Asian J Pharm.

[97] Salama RAA, Bader KN, Rahman AS, Hashmi FY (2018). Parents knowledge, attitudes and practice of use of antibiotics for upper respiratory tract infections in children: a cross-sectional study in ras al khaimah, United Arab Emirates. Epidemiol Biostat Public Health.

[98] Aziz MM, Haider F, Rasool MF, Hashmi FK, Bahsir S, Li P, Zhao M, Alshammary TM, Fang Y (2021). Dispensing of non-prescribed antibiotics from community pharmacies of Pakistan: a cross-sectional survey of pharmacy staff’s opinion. Antibiotics.

[99] Hicks JP, Latham SM, Huque R, Das M, Newell J, Abdullah S, Al Azdi Z, Jahan I, Rassi C, Hamade P (2021). Antibiotic practices among household members and their domestic animals within rural communities in Cumilla district, Bangladesh: a cross-sectional survey. BMC Public Health.

[100] Roien R, Bhandari D, Hosseini SMR, Mosawi SH, Ataie MA, Ozaki A, Martellucci CA, Kotera Y, Delshad MH, Sawano T (2021). Prevalence and determinants of self-medication with antibiotics among general population in Afghanistan. Expert Rev Anti Infect Ther.

[101] Allwell-Brown G, Hussain-Alkhateeb L, Kitutu FE, Strömdahl S, Mårtensson A, Johansson EW (2020). Trends in reported antibiotic use among children under 5 years of age with fever, diarrhoea, or cough with fast or difficult breathing across low-income and middle-income countries in 2005–17: a systematic analysis of 132 national surveys from 73 countries. Lancet Glob Health.

[102] Ngocho JS, Horumpende PG, de Jonge MI, Mmbaga BT (2020). Inappropriate treatment of community-acquired pneumonia among children under five years of age in Tanzania. IJID.

[103] Hijazi K, Joshi C, Gould IM (2019). Challenges and opportunities for antimicrobial stewardship in resource-rich and resource-limited countries. Expert Rev Anti Infect Ther.

[104] Davido B, Bouchand F, Calin R, Makhloufi S, Lagrange A, Senard O, Perronne C, Villart M, Salomon J, Dinh A (2016). High rates of off-label use in antibiotic prescriptions in a context of dramatic resistance increase: a prospective study in a tertiary hospital. Int J Antimicrob Agents.

[105] Elbasha EH (2003). Deadweight loss of bacterial resistance due to overtreatment. Health Econ.

[106] Marzan M, Islam DZ, Lugova H, Krishnapillai A, Haque M, Islam S (2021). Knowledge, attitudes, and practices of antimicrobial uses and resistance among public university students in Bangladesh. Infect Drug Resist.

[107] Williams P, Cotta MO, Roberts JA (2019). Pharmacokinetics/pharmacodynamics of β-lactams and therapeutic drug monitoring: from theory to practical issues in the intensive care unit. Semin Respir Crit Care Med.

[108] Odenholt I, Gustafsson I, Löwdin E, Cars O (2003). Suboptimal antibiotic dosage as a risk factor for selection of penicillin-resistant Streptococcus pneumoniae: in vitro kinetic model. Antimicrob Agents Chemother.

[109] Gu J, Zhao J, Huang Y, Yang W, Ren Z, Li W, Fan Y, Zhang Q, Zhang F, Fu Y (2015). Use of antibiotics by urban and rural residents in Heilongjiang Province, China: cross-sectional study. TMIH.

[110] Kenyon C, Manoharan-Basil SS (2020). Cultural drivers of antibiotic consumption in high-income countries: a global ecological analysis. Microb Drug Resist (Larchmont, NY).

[111] Deschepper R, Grigoryan L, Lundborg CS, Hofstede G, Cohen J, Kelen GV, Deliens L, Haaijer-Ruskamp FM (2008). Are cultural dimensions relevant for explaining cross-national differences in antibiotic use in Europe?. BMC Health Serv Res.

[112] Kopsidas I, Vergnano S, Spyridis N, Zaoutis T, Patel S (2020). A survey on national pediatric antibiotic stewardship programs, networks and guidelines in 23 European countries. Pediatr Infect Dis J.

[113] Yaya S, Bishwajit G (2019). Burden of acute respiratory infections among under-five children in relation to household wealth and socioeconomic status in Bangladesh. Tro. Med Infect Dis.

[114] Hanifi SM, Mahmood SS, Bhuiya A (2014). Cause-specific mortality and socioeconomic status in Chakaria, Bangladesh. Glob Health Action.

[115] Mahumud RA, Alam K, Renzaho AMN, Sarker AR, Sultana M, Sheikh N, Rawal LB, Gow J (2019). Changes in inequality of childhood morbidity in Bangladesh 1993–2014: a decomposition analysis. PLoS ONE.

[116] Rheingans R, Anderson JDT, Bagamian KH, Laytner LA, Pecenka CJ, Gilani SSA, Ahmed M (2018). Effects of geographic and economic heterogeneity on the burden of rotavirus diarrhea and the impact and cost-effectiveness of vaccination in Pakistan. Vaccine.

[117] Walker CLF, Rudan I, Liu L, Nair H, Theodoratou E, Bhutta ZA, O'Brien KL, Campbell H, Black RE (2013). Global burden of childhood pneumonia and diarrhoea. Lancet (London, England).

[118] Glass RI, Bresee JS, Turcios R, Fischer TK, Parashar UD, Steele AD (2005). Rotavirus vaccines: targeting the developing world. J Infect Dis.

[119] He Z, Bishwajit G, Zou D, Yaya S, Cheng Z, Zhou Y (2018). Burden of common childhood diseases in relation to improved water, sanitation, and hygiene (WASH) among Nigerian children. Int J Environ Res Public Health.

[120] Bhutta ZA (2008). Drug resistant infections in poor countries: a major burden on children. BMJ.

[121] Murray CJ, Lopez AD (1997). Global mortality, disability, and the contribution of risk factors: Global Burden of Disease Study. Lancet (London, England).

[122] Olaru ID, Kibuule D, Godman B (2020). Implications of antibiotic exposure among children in low-income and middle income countries. Lancet Infect Dis.

[123] Zeru T, Berihu H, Buruh G, Gebrehiwot H, Zeru M (2020). Parental knowledge and practice on antibiotic use for upper respiratory tract infections in children, in aksum town health institutions, northern Ethiopia: a cross-sectional study. Pan Afr Med J.

[124] Mboya EA, Davies ML, Horumpende PG, Ngocho JS (2020). Inadequate knowledge on appropriate antibiotics use among clients in the Moshi municipality Northern Tanzania. PLoS ONE.

[125] Mahase E: Non-prescription antibiotic use is “public health problem” in US, finds study. In: vol. 366. British Medical Journal Publishing Group; 2019: Accessed February 27, 2022. 10.1136/bmj.l4836. 10.1136/bmj.l4836 31340928

[126] Abdelaziz AI, Tawfik AG, Rabie KA, Omran M, Hussein M, Abou-Ali A (2019). Quality of community pharmacy practice in antibiotic self-medication encounters: a simulated patient study in upper Egypt. Antibiotics.

[127] Madden JM, Quick JD, Ross-Degnan D, Kafle KK (1997). Undercover careseekers: simulated clients in the study of health provider behavior in developing countries. Soc Sci Med.

[128] Schoenthaler A, Albright G, Hibbard J, Goldman R (2017). Simulated conversations with virtual humans to improve patient-provider communication and reduce unnecessary prescriptions for antibiotics: a repeated measure pilot study. JMIR Med Educ.

[129] Mate I, Come CE, Gonçalves MP, Cliff J, Gudo ES (2019). Knowledge, attitudes and practices regarding antibiotic use in Maputo City Mozambique. PLoS ONE.

[130] Wang X, Lin L, Xuan Z, Li L, Zhou X (2018). Keeping antibiotics at home promotes self-medication with antibiotics among Chinese university students. Int J Environ Res Public Health.

[131] Auta A, Hadi M, Oga E, Adewuyi E, Abdu-Aguye S, Adeloye D, Strickland-Hodge B, Morgan D (2018). Global access to antibiotics without prescription in community pharmacies: a systematic review and meta-analysis. J Infect.

[132] Alhomoud F, Aljamea Z, Almahasnah R, Alkhalifah K, Basalelah L, Alhomoud FK (2017). Self-medication and self-prescription with antibiotics in the Middle East—do they really happen? A systematic review of the prevalence, possible reasons, and outcomes. Int J Infect Dis.

[133] Om C, Daily F, Vlieghe E, McLaughlin JC, McLaws M-L (2017). Pervasive antibiotic misuse in the Cambodian community: antibiotic-seeking behaviour with unrestricted access. Antimicrob Resist Infect Control.

[134] Cortez J, Rosário E, Pires JE, Taborda Lopes J, Francisco M, Vlieghe E, Brito M (2017). Antimicrobial storage and antibiotic knowledge in the community: a cross-sectional pilot study in north-western Angola. Int J Infect Dis.

[135] Barker AK, Brown K, Ahsan M, Sengupta S, Safdar N (2017). What drives inappropriate antibiotic dispensing? A mixed-methods study of pharmacy employee perspectives in Haryana India. BMJ Open.

[136] Barker AK, Brown K, Ahsan M, Sengupta S, Safdar N (2017). Social determinants of antibiotic misuse: a qualitative study of community members in Haryana, India. BMC Public Health.

[137] Dobrescu A, Nussbaumer-Streit B, Klerings I, Wagner G, Persad E, Sommer I, Herkner H, Gartlehner G (2021). Restricting evidence syntheses of interventions to English-language publications is a viable methodological shortcut for most medical topics: a systematic review. J Clin Epidemiol.

[138] Jüni P, Holenstein F, Sterne J, Bartlett C, Egger M (2002). Direction and impact of language bias in meta-analyses of controlled trials: empirical study. Int J Epidemiol.

[139] Moher D, Pham B, Lawson ML, Klassen TP (2003). The inclusion of reports of randomised trials published in languages other than English in systematic reviews. Health Technol Assess.

[140] Morrison A, Polisena J, Husereau D, Moulton K, Clark M, Fiander M, Mierzwinski-Urban M, Clifford T, Hutton B, Rabb D (2012). The effect of English-language restriction on systematic review-based meta-analyses: a systematic review of empirical studies. Int J Technol Assess Health Care.

[141] Pham B, Klassen TP, Lawson ML, Moher D (2005). Language of publication restrictions in systematic reviews gave different results depending on whether the intervention was conventional or complementary. J Clin Epidemiol.

