# Supplementary material for: Non-prescribed antibiotic use for children at community levels in low- and middle-income countries: a systematic review and meta-analysis
Source: J Pharm Policy Pract. 2022 Sep 30;15:57. doi: 10.1186/s40545-022-00454-8 (PMC9524137; doi:10.1186/s40545-022-00454-8)
Supplement: Supplementary file 2 — Additional file 2. Methodological quality assessment of individual studies. [file 40545_2022_454_MOESM2_ESM.docx]

**Additional file 2. Methodological quality assessment for the included studies, July 2022.**

| **Studies** | **JBI’s appraisal checklist for studies reporting prevalence data** | | | | | | | | |
| --- | --- | --- | --- | --- | --- | --- | --- | --- | --- |
|  | **Q1** | **Q2** | **Q3** | **Q4** | **Q5** | **Q6** | **Q7** | **Q8** | **Q9** |
| Abegaz T et al, 2016 | Yes | No | No | Yes | Yes | Yes | Yes | Yes | UC |
| Adeyemi OO et al, 2021 | No | No | Yes | Yes | Yes | Yes | Yes | Yes | Yes |
| Al-Noman MS and Elnimeiri MK, 2022 | Yes | Yes | Yes | Yes | Yes | No | No | Yes | Yes |
| Al-Shawi M et al, 2018 | Yes | Yes | Yes | Yes | Yes | Yes | No | Yes | No |
| Chang J et al, 2016 | Yes | Yes | Yes | Yes | Yes | Yes | Yes | Yes | Yes |
| Chang J et al, 2018 | Yes | Yes | Yes | No | Yes | Yes | Yes | Yes | No |
| Chang J et al, 2019 | Yes | Yes | Yes | Yes | Yes | Yes | Yes | Yes | Yes |
| Diwan V et al, 2015 | Yes | UC | No | Yes | Yes | Yes | Yes | No | Yes |
| Edessa D et al, 2022 | No | Yes | No | Yes | Yes | Yes | No | Yes | Yes |
| Hallit S et al, 2020 | Yes | Yes | No | Yes | Yes | Yes | Yes | Yes | Yes |
| Hussain A et al, 2012 | Yes | Yes | Yes | Yes | Yes | Yes | Yes | Yes | Yes |
| Kibuule et al, 2016 | Yes | No | UC | No | Yes | Yes | Yes | Yes | Yes |
| Koji E et al, 2019 | Yes | Yes | Yes | Yes | Yes | Yes | No | Yes | Yes |
| Lanyero H et al, 2020 | Yes | Yes | Yes | Yes | Yes | Yes | No | Yes | Yes |
| Lanyero H et al, 2021 | Yes | Yes | No | Yes | Yes | Yes | No | Yes | Yes |
| Lin L et al, 2020 | Yes | Yes | Yes | Yes | Yes | Yes | No | Yes | Yes |
| Lin L et al, 2021 | Yes | Yes | Yes | Yes | Yes | Yes | No | Yes | Yes |
| Malik U et al, 2021 | Yes | Yes | Yes | Yes | Yes | Yes | Yes | Yes | Yes |
| Miyazaki A et al, 2020 | Yes | UC | No | Yes | Yes | Yes | Yes | No | Yes |
| Mukattash T et al, 2020 | Yes | Yes | Yes | No | Yes | Yes | Yes | Yes | Yes |
| Nyeko R et al, 2022 | Yes | Yes | No | Yes | Yes | Yes | No | Yes | UC |
| Ocan M et al, 2017 | Yes | Yes | Yes | Yes | No | Yes | Yes | Yes | Yes |
| Ogbo P et al, 2014 | Yes | Yes | Yes | No | No | Yes | Yes | No | Yes |
| Paredes JL et al, 2022 | No | Yes | No | Yes | Yes | No | Yes | Yes | Yes |
| Saengcharoen W et al, 2010 | Yes | Yes | No | No | Yes | Yes | Yes | No | Yes |
| Samir N et al, 2021 | Yes | Yes | Yes | Yes | Yes | Yes | Yes | Yes | Yes |
| Shet A et al, 2015 | Yes | Yes | No | No | Yes | Yes | Yes | Yes | Yes |
| Shi L et al, 2020 | Yes | UC | No | Yes | Yes | Yes | Yes | Yes | Yes |
| Simon B et al, 2020 | Yes | Yes | Yes | Yes | Yes | Yes | No | Yes | Yes |
| Sun C et al, 2019 | Yes | Yes | Yes | Yes | Yes | Yes | Yes | Yes | No |
| Togoobaatar G et al, 2010 | Yes | Yes | Yes | Yes | Yes | Yes | No | Yes | Yes |
| Wu J et al, 2021 | Yes | Yes | Yes | Yes | Yes | Yes | No | Yes | Yes |
| Xu J et al, 2020 | Yes | Yes | Yes | Yes | Yes | Yes | Yes | Yes | Yes |
| Xu Y et al, 2020 | Yes | Yes | Yes | Yes | Yes | Yes | No | Yes | Yes |
| Yu M et al, 2014 | Yes | Yes | Yes | Yes | Yes | Yes | No | Yes | Yes |
| Yuan J et al, 2022 | Yes | Yes | Yes | Yes | No | Yes | No | Yes | Yes |
| Zawahir S et al, 2022 | Yes | Yes | Yes | Yes | Yes | Yes | Yes | Yes | UC |
| Zhu Y et al, 2021 | Yes | Yes | Yes | Yes | Yes | Yes | Yes | Yes | Yes |
| Zwisler G et al, 2013 | Yes | No | No | Yes | Yes | Yes | Yes | Yes | UC |

**Note:** UC, unclear; and Q1-9, JBI’s critical appraisal checklist for studies reporting prevalence data {Q1: Was the sample frame appropriate to address the target population? Q2: Were study participants sampled in an appropriate way? Q3: Was the sample size adequate? Q4: Were the study subjects and the setting described in detail? Q5: Was the data analysis conducted with sufficient coverage of the identified sample? Q6: Were valid methods used for the identification of the condition? Q7: Was the condition measured in a standard, reliable way for all participants? Q8: Was there appropriate statistical analysis? Q9: Was the response rate adequate, and if not, was the low response rate managed appropriately?}.
