# Supplementary material for: Non-prescribed antibiotic use for children at community levels in low- and middle-income countries: a systematic review and meta-analysis
Source: J Pharm Policy Pract. 2022 Sep 30;15:57. doi: 10.1186/s40545-022-00454-8 (PMC9524137; doi:10.1186/s40545-022-00454-8)
Supplement: Supplementary file 3 — Additional file 3. Risk of bias assessed for the included individual studies. [file 40545_2022_454_MOESM3_ESM.docx]

**S3 Table. Risk-of-bias assessment across the individual studies.**

| **Study** | **Domains** | | | | **Overall judgement** | **Items** | | | | | | | | | | **Overall score** |
| --- | --- | --- | --- | --- | --- | --- | --- | --- | --- | --- | --- | --- | --- | --- | --- | --- |
|  | **D1** | **D2** | **D3** | **D4** |  | **Q1** | **Q2** | **Q3** | **Q4** | **Q5** | **Q6** | **Q7** | **Q8** | **Q9** | **Q10** |  |
| Abegaz T et al, 2016 | High | Low | Low | Low | Low | 1 | 1 | 1 | 0 | 0 | 0 | 0 | 0 | 0 | 0 | 3 |
| Adeyemi OO et al, 2021 | Some concerns | Low | Low | Low | Low | 0 | 1 | 1 | 0 | 0 | 0 | 1 | 0 | 0 | 0 | 3 |
| Al-Noman MS and Elnimeiri MK, 2022 | Some concerns | Low | Some concerns | Low | Some concerns | 1 | 1 | 0 | 0 | 0 | 1 | 1 | 0 | 0 | 0 | 4 |
| Al-Shawi M et al, 2018 | Some concerns | High | Some concerns | Low | Some concerns | 1 | 0 | 0 | 1 | 1 | 1 | 1 | 0 | 0 | 0 | 5 |
| Chang J et al, 2017 | High | Low | Low | Low | Low | 1 | 1 | 1 | 0 | 0 | 0 | 0 | 0 | 0 | 0 | 3 |
| Chang J et al, 2018 | Low | High | Low | Low | Low | 1 | 0 | 0 | 0 | 1 | 0 | 0 | 0 | 0 | 0 | 2 |
| Chang J et al, 2019 | Some concerns | Low | Low | Low | Low | 1 | 0 | 1 | 0 | 0 | 0 | 0 | 0 | 0 | 0 | 2 |
| Diwan V et al, 2015 | High | Low | Low | Low | Low | 1 | 1 | 1 | 0 | 0 | 0 | 0 | 0 | 0 | 0 | 3 |
| Edessa D et al, 2022 | Some concerns | Low | Low | Low | Some concerns | 1 | 0 | 1 | 0 | 1 | 1 | 0 | 0 | 0 | 0 | 4 |
| Hallit S et al, 2018 | High | Low | Low | Low | Low | 1 | 1 | 1 | 1 | 0 | 0 | 0 | 1 | 0 | 0 | 5 |
| Hussain A et al, 2012 | Some concerns | Low | Low | Low | Low | 1 | 0 | 1 | 0 | 0 | 0 | 0 | 0 | 0 | 0 | 2 |
| Kibuule D et al, 2016 | Some concerns | High | Low | Low | Low | 1 | 1 | 0 | 0 | 1 | 0 | 0 | 0 | 0 | 0 | 3 |
| Koji E et al, 2019 | Some concerns | Low | Some concerns | Low | Some concerns | 1 | 1 | 0 | 0 | 0 | 1 | 0 | 1 | 0 | 0 | 4 |
| Lanyero H et al, 2020 | Some concerns | High | Low | Low | Low | 1 | 0 | 1 | 0 | 1 | 0 | 0 | 0 | 0 | 0 | 3 |
| Lanyero H et al, 2021 | Some concerns | High | Low | High | Some concerns | 1 | 0 | 1 | 0 | 1 | 0 | 0 | 0 | 0 | 1 | 4 |
| Lin L et al, 2020 | Low | High | Some concerns | High | Some concerns | 0 | 0 | 0 | 1 | 1 | 1 | 1 | 0 | 0 | 1 | 5 |
| Lin L et al, 2021 | Low | High | Low | High | Low | 0 | 0 | 1 | 0 | 1 | 0 | 0 | 0 | 0 | 1 | 3 |
| Malik U et al, 2021 | Some concerns | High | Low | Low | Low | 1 | 1 | 0 | 0 | 1 | 0 | 0 | 0 | 0 | 0 | 3 |
| Miyazaki A et al, 2020 | Some concerns | High | Low | Low | Some concerns | 1 | 0 | 1 | 0 | 1 | 1 | 0 | 0 | 0 | 0 | 4 |
| Mukattash T et al, 2019 | High | High | Low | Low | Some concerns | 1 | 1 | 1 | 0 | 1 | 0 | 0 | 0 | 0 | 0 | 4 |
| Nyeko R et al, 2022 | Some concerns | Low | Some concerns | High | Some concerns | 1 | 1 | 0 | 0 | 0 | 1 | 1 | 0 | 0 | 1 | 5 |
| Ocan M et al, 2017 | Low | High | Low | Low | Low | 1 | 0 | 0 | 0 | 1 | 0 | 0 | 0 | 0 | 0 | 2 |
| Ogbo P et al, 2014 | High | Low | Low | Low | Low | 1 | 1 | 1 | 0 | 0 | 0 | 0 | 0 | 0 | 0 | 3 |
| Paredes JL et al, 2022 | High | High | Low | Low | Some concerns | 1 | 0 | 1 | 1 | 1 | 1 | 0 | 0 | 1 | 0 | 6 |
| Saengcharoen W et al, 2010 | Low | Low | Low | Low | Low | 1 | 0 | 0 | 0 | 0 | 0 | 1 | 0 | 0 | 0 | 2 |
| Samir N et al, 2021 | Low | High | Low | High | Low | 0 | 0 | 0 | 0 | 1 | 0 | 0 | 0 | 0 | 1 | 2 |
| Shet A et al, 2015 | Low | Low | Low | Low | Low | 1 | 0 | 0 | 0 | 0 | 0 | 0 | 0 | 0 | 0 | 1 |
| Shi L et al, 2020 | Some concerns | Low | Low | High | Low | 1 | 0 | 1 | 0 | 0 | 0 | 0 | 0 | 0 | 1 | 3 |
| Simon B et al, 2020 | Low | High | Low | Low | Low | 1 | 0 | 0 | 0 | 1 | 0 | 0 | 0 | 0 | 0 | 2 |
| Sun C et al, 2019 | Low | High | Low | Low | Low | 0 | 0 | 0 | 0 | 1 | 0 | 0 | 0 | 0 | 0 | 1 |
| Togoobaatar G et al, 2010 | Low | High | Low | Low | Low | 1 | 0 | 0 | 0 | 1 | 0 | 0 | 0 | 0 | 0 | 2 |
| Wu J et al, 2021 | Low | High | Low | Low | Low | 1 | 0 | 0 | 0 | 1 | 0 | 0 | 0 | 0 | 0 | 2 |
| Xu J et al, 2020 | Low | High | Low | Low | Low | 1 | 0 | 0 | 0 | 1 | 0 | 0 | 0 | 0 | 0 | 2 |
| Xu Y et al, 2020 | Low | High | Low | Low | Low | 0 | 0 | 0 | 0 | 1 | 0 | 0 | 0 | 0 | 0 | 1 |
| Yu M et al, 2014 | High | High | Some concerns | High | High | 1 | 1 | 1 | 0 | 1 | 1 | 1 | 0 | 0 | 1 | 7 |
| Yuan J et al, 2022 | Low | Low | Some concerns | Low | Low | 0 | 0 | 0 | 1 | 0 | 1 | 1 | 0 | 0 | 0 | 3 |
| Zawahir S et al, 2022 | Low | Low | Low | Low | Low | 0 | 0 | 1 | 0 | 0 | 0 | 0 | 0 | 0 | 0 | 1 |
| Zhu Y et al, 2021 | High | High | Some concerns | High | High | 1 | 1 | 0 | 1 | 1 | 1 | 1 | 0 | 0 | 1 | 7 |
| Zwisler G et al, 2013 | Some concerns | High | Low | High | Some concerns | 1 | 1 | 0 | 0 | 1 | 0 | 0 | 0 | 0 | 1 | 4 |
| **Note**:   1. The ten items adapted from Hoy et al tool used to assess risk-of-bias for prevalence studies were classified into four domains. D1: bias arising from the study participant selection process (assessed with items 1-4); D2: bias linked to data collection process (assessed with item 5); D3: bias in measurement of the outcome (assessed with items 6-9); and D4: bias due to statistics parameter (assessed with item 10). 2. The ten appraisal items included Q1: Was the study’s target population a close representation of the national population in relation to relevant variables? Q2: Was the sampling frame a true or close representation of the target population? Q3: Was some form of random selection used to select the sample, OR, was a census undertaken? Q4: Was the likelihood of nonresponse bias minimal? Q5: Were data collected directly from the subjects (as opposed to a proxy)? Q6: Was an acceptable case definition used in the study? Q7: Was the study instrument that measured the parameter of interest shown to have validity and reliability? Q8: Was the same mode of data collection used for all subjects? Q9: Was the length of the shortest prevalence period for the parameter of interest appropriate? Q10: Were the numerator(s) and denominator(s) for the parameter of interest appropriate? 3. Response for each of the appraisal item was scored as ‘1’ for ‘no’ and as ‘0’ for ‘yes’ answers. 4. The overall risk-of-bias level was rated as low risk (for 0-3 scores); some concerns (for 4-6 scores); and high risk (for 7-10 scores) based on the summary of rates scored. | | | | | | | | | | | | | | | | |
