# Supplementary figures and images for: Non-prescribed antibiotic use for children at community levels in low- and middle-income countries: a systematic review and meta-analysis
Source: J Pharm Policy Pract. 2022 Sep 30;15:57. doi: 10.1186/s40545-022-00454-8 (PMC9524137; doi:10.1186/s40545-022-00454-8)

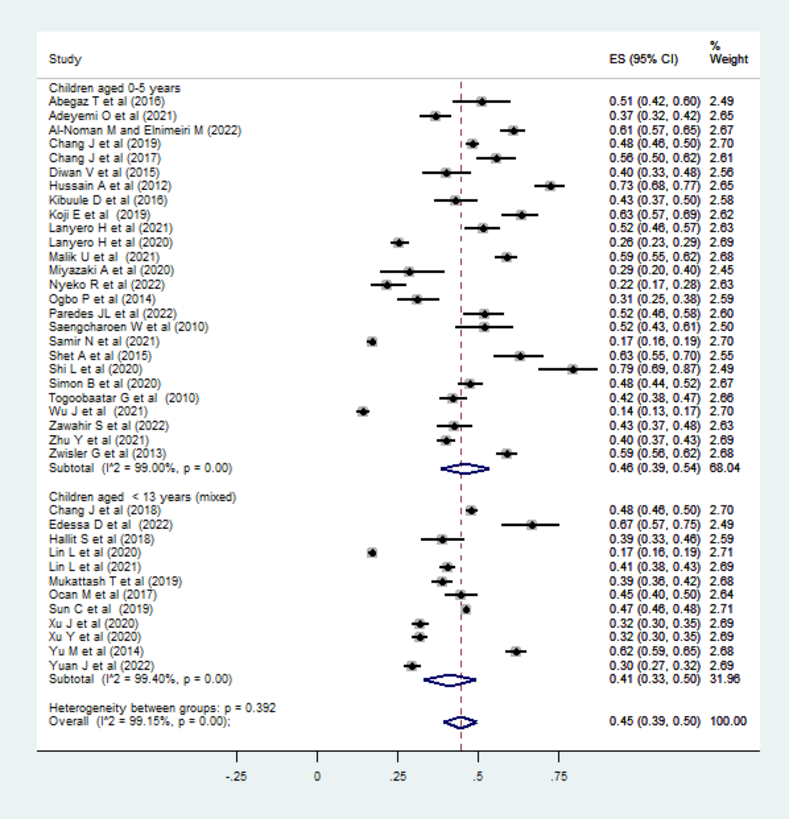

Supplement: Supplementary file 4 — Additional file 4. Nonprescription antibiotic use for children by region. [file 40545_2022_454_MOESM4_ESM.tif]

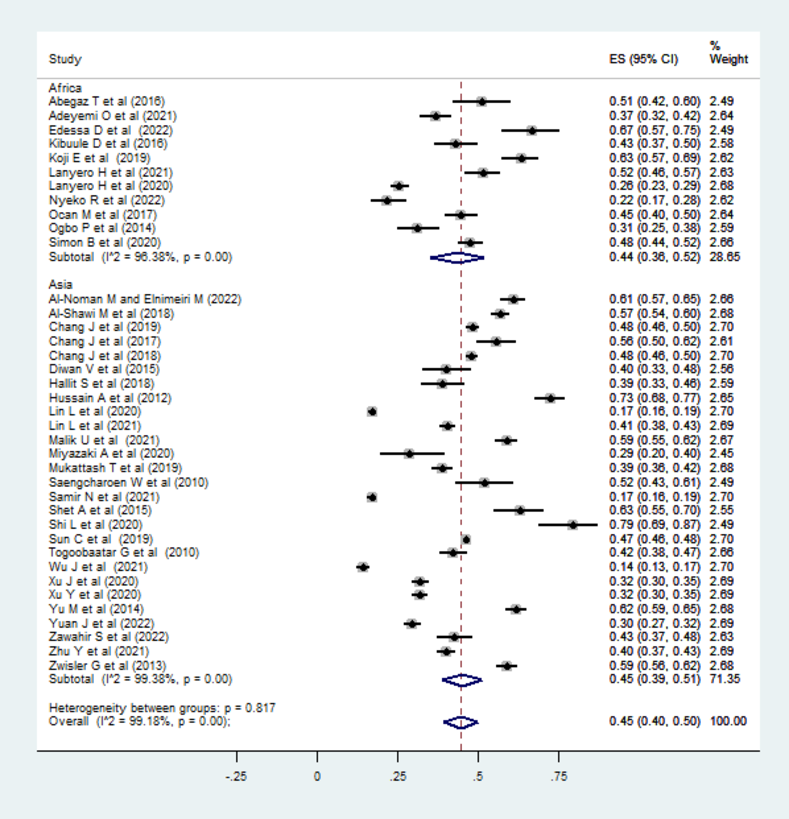

Supplement: Supplementary file 5 — Additional file 5. Nonprescription antibiotic use for children by the children’s age. [file 40545_2022_454_MOESM5_ESM.tif]

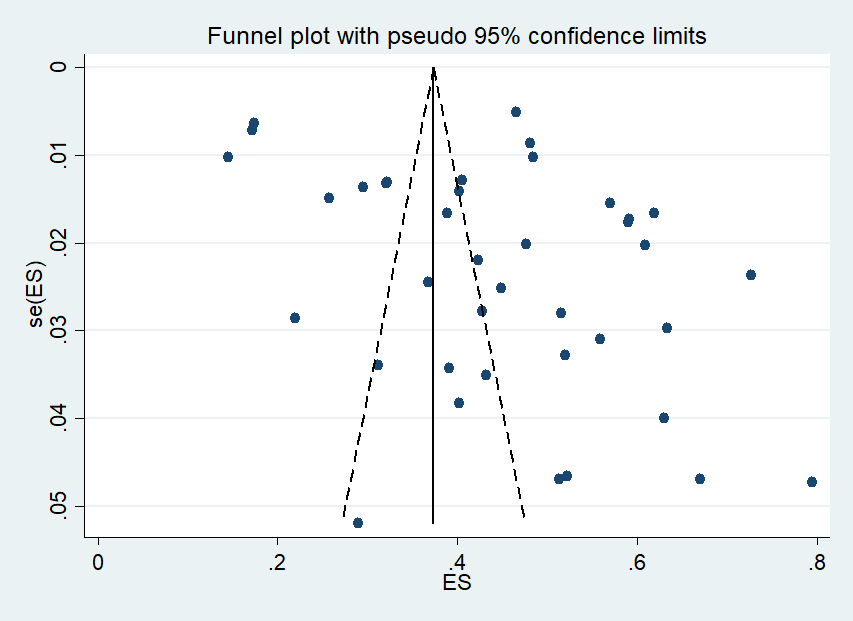

Supplement: Supplementary file 6 — Additional file 6. Funnel plot for publication bias. [file 40545_2022_454_MOESM6_ESM.tif]
